# Multi-Omics Reveals Dose-Dependent Modulation of Intestinal Lipid Metabolism and Metabolic Inflammation by 1-Deoxynojirimycin in Amur Sturgeon (*Acipenser schrenckii*)

**DOI:** 10.3390/biology15201809

**Published:** 2026-10-10

**Authors:** Nan Zhang, Wanwan Zhu, Xuekai Wang, Yi Xiong, Fuyu Yang

**Affiliations:** 1 Department of Grassland Science, College of Animal Science, Guizhou University, Guiyang 550025, China; zn2580042898@126.com (N.Z.); wan850793534@126.com (W.Z.); 2 Beijing Tianyuanyugang Farm Co., Ltd. (Beijing Amur Sturgeon Elite Breeding Base), Beijing 101400, China; 3 College of Grassland Science and Technology, China Agricultural University, Beijing 100193, China; xkwang2016@163.com; 4 Frontier Technology Research Institute of China Agricultural University in Shenzhen, Shenzhen 518000, China; xiongleslie@126.com

**Keywords:** 1-deoxynojirimycin, *Acipenser schrenckii*, lipid metabolism, metabolic inflammation, multi-omics, dose–response, functional additive

## Abstract

High-lipid diets in intensive sturgeon aquaculture induce lipid metabolic disorders and metabolic inflammation. 1-Deoxynojirimycin (DNJ), a mulberry-derived α-glucosidase inhibitor, shows lipid-regulating and anti-inflammatory activities, but its dose-dependent effects in basal actinopterygians such as sturgeon remain unexplored. Juvenile Amur sturgeons (*Acipenser schrenckii*) were fed graded DNJ levels (0, 5, 10, and 15 mg/kg) for 8 weeks. Integrating serum biochemistry, transcriptomics, untargeted metabolomics, 16S rRNA sequencing, and qRT-PCR, we found that DNJ10 (10 mg/kg) most effectively reduced serum triglyceride, total cholesterol, glucose, and malondialdehyde, upregulated intestinal *elovl2*, and left *acsl4* showing a consistent downward trend.. Gut microbiota analysis revealed that DNJ10 enriched Ruminococcaceae and *Paludicola*, associated with butyrate production and lipid hydrolysis, whereas DNJ15 shifted the community toward bile-metabolizing spirochetes with reduced anti-inflammatory capacity. Transcriptome–metabolome correlation and qRT-PCR confirmed that DNJ10 simultaneously suppressed NF-κB-mediated inflammatory signaling and enhanced intestinal fatty acid elongation and lipid turnover (elovl2, enpp7). Quadratic regression identified an optimal DNJ level of 8.0–8.5 mg/kg. These findings demonstrate that low-to-medium doses of DNJ effectively regulate lipid metabolism and metabolic inflammation via the gut–liver axis, whereas the anti-inflammatory effect diminishes and is partly reversed at the high dose (DNJ15). DNJ10 (10 mg/kg) was the experimental dose closest to the regression-estimated optimum of 8.0–8.5 mg/kg, which is the dosage window recommended for practical application in sturgeon aquaculture. These findings provide a theoretical basis for the application of DNJ as a functional additive in high-lipid intensive aquaculture.

## Simple Summary

Sturgeons are ancient “living fossil” fish that are highly valued for caviar and meat, but intensive farming with high-fat feeds can cause health problems such as excess fat accumulation and inflammation. In this study, we tested whether 1-deoxynojirimycin (DNJ), a natural compound extracted from mulberry leaves, could improve the health of farmed Amur sturgeon. We fed sturgeon with four different amounts of DNJ for 8 weeks and examined their blood, gut tissue, gut microbes, and metabolism. We found that a moderate dose of DNJ (10 mg/kg) worked best: it reduced blood fat and blood sugar, alleviated oxidative stress in the gut, and helped the gut grow beneficial bacteria that produce health-promoting substances. A higher dose was less effective, and a lower dose was too mild. Our results suggest that adding the right amount of DNJ to sturgeon feed could improve fish health and support more sustainable aquaculture.

## 1. Introduction

Sturgeons (order Acipenseriformes), whose earliest known fossil record dates back to approximately 200 million years ago in the Jurassic period of the Mesozoic era, are widely acclaimed as “living fossils in water.” As key model organisms for deciphering the origin and evolutionary history of vertebrates, they possess exceptional scientific value in paleobiology and comparative physiology [1,2]. The sturgeon industry also offers prominent economic advantages: caviar, its primary processed product, is a high-end luxury aquatic commodity with substantial market added value, while fresh sturgeon meat commands significantly higher prices than conventional farmed fish, underscoring the industry’s considerable profit potential [3]. However, driven by chronic overfishing, natural habitat degradation, and water pollution, wild sturgeon resources have continued to decline, making large-scale intensive aquaculture the core pathway for ensuring the sustainable development of the sturgeon industry [4]. China is the world’s leading sturgeon-farming and caviar-exporting country; in 2023, its domestic sturgeon aquaculture output reached 149,400 tonnes, with caviar exports totaling 275.83 tonnes, ranking first globally in industry scale (latest official data from the Ministry of Agriculture and Rural Affairs of China; no newer official statistics are available) [5,6].

Feed expenditure accounts for 50–60% of the total cost of intensive aquaculture, while prices of high-quality animal protein ingredients such as fishmeal have long remained volatile at elevated levels, continuously squeezing profit margins [7]. To reduce formulation costs and shorten the culture cycle, commercial compound feeds commonly increase lipid inclusion. Lipids provide high-density energy, exert a protein-sparing effect that reduces reliance on expensive fishmeal, and simultaneously accelerate fish growth, thereby achieving cost reduction and efficiency gains in aquaculture [8]. Nevertheless, long-term feeding of high-lipid diets disrupts lipid metabolic homeostasis in fish, triggering a cascade of health problems including reduced feed intake, growth retardation, suppressed immune function, and exacerbated oxidative stress [9,10]. For farmed sturgeon whose core output is caviar, excessive lipid accumulation in the body can also directly impair the spawning performance of broodstock, further constraining the economic efficiency of the sturgeon industry [11].

1-Deoxynojirimycin (DNJ), a naturally occurring polyhydroxylated alkaloid derived from mulberry leaves, is a potent α-glucosidase inhibitor with multifaceted bioactivities in regulating glucose and lipid metabolism, alleviating oxidative damage, and balancing immune homeostasis [12]. Accumulating evidence has demonstrated that DNJ significantly lowers total cholesterol (TC) and triglyceride (TG) levels and ameliorates lipid metabolic disorders via the IRS1/PI3K/AKT signaling pathway [13]. It also reduces lipid accumulation, pro-inflammatory cytokine levels, and the protein expression of fatty acid synthase (FAS) and peroxisome proliferator-activated receptor γ (PPAR-γ) in palmitic acid (PA)-induced fatty liver cells [14]. Meanwhile, as the principal anti-inflammatory component of *Morus alba* L. (Sangzhi) alkaloids (SZ-A), DNJ attenuates inflammation by blocking the activation of the p38 MAPK, ERK, and JNK signaling pathways [15]. DNJ treatment markedly decreases intracellular malondialdehyde (MDA) content, elevates the activities of total superoxide dismutase (T-SOD) and catalase (CAT), upregulates *Nrf2* mRNA expression, and downregulates the expression of inflammatory cytokine-related genes, thereby ameliorating oxidative stress and inflammatory responses in the chicken intestine [16,17]. Furthermore, studies in the aquatic amphibian model organism *Andrias davidianus* (Chinese giant salamander) have confirmed that DNJ significantly improves growth performance, intestinal digestive and absorptive function, antioxidant capacity, and immune competence, while effectively optimizing gut microbiota structure and enhancing microbial diversity [18].

Despite these promising findings in mammals and amphibians, our understanding of DNJ’s metabolic actions in fish remains largely unexplored. The gut–liver axis (GLA), a bidirectional communication network linking intestinal microbiota, their metabolites (bile acids, short-chain fatty acids, and endotoxins), and host immune-metabolic responses, represents a central hub for lipid homeostasis and inflammation [19]. In teleosts, dietary interventions that perturb the GLA have been reported to profoundly influence host lipid deposition via microbiota-derived signaling molecules that modulate host farnesoid X receptor (FXR) and G protein-coupled bile acid receptor (TGR5) pathways [20]. Recent multi-omics investigations in fish have established robust association patterns between gut microbial community shifts and host metabolic reprogramming, providing a methodological framework for dissecting GLA regulatory networks in non-mammalian vertebrates [21]. However, whether these GLA-mediated mechanisms apply to DNJ—and how they operate in sturgeon, a species with distinct gut architecture and microbial ecology—has not been systematically investigated. Moreover, previous aquaculture studies employing mulberry leaf extracts, which contain complex mixtures of polyphenols and flavonoids alongside DNJ, have failed to attribute observed phenotypic improvements unambiguously to DNJ itself, and have largely been confined to conventional growth and serum biochemistry endpoints, without the concurrent integration of the gut transcriptome, gut metabolome, and gut microbiota that is required to resolve the underlying regulatory networks—an integrated multi-omics interrogation that remains essentially absent in sturgeon and other non-model aquaculture species [22,23]. Consequently, the dose-dependent regulatory mechanisms of DNJ on lipid metabolism, metabolic inflammation, and gut–liver axis interactions in sturgeon remain unclear, and the optimal dietary inclusion level has not been quantitatively defined.

To address these knowledge gaps, the present study employed a multi-tiered integrative approach using *Acipenser schrenckii* (Amur sturgeon), a representative acipenserid species of commercial and evolutionary significance. We designed three graded dietary DNJ inclusion levels and systematically evaluated: (i) dose-dependent effects on growth performance and systemic metabolic profiles; (ii) intestinal transcriptomic signatures; (iii) untargeted serum metabolomics; (iv) 16S rRNA sequencing of intestinal microbiota; and (v) targeted qPCR validation of key genes. By integrating these layers of biological information, we aim to construct a holistic picture of the DNJ-mediated GLA regulatory landscape in sturgeon. Specifically, we hypothesize that DNJ ameliorates high-lipid-diet-induced lipid metabolic disorder and metabolic inflammation through a coordinated mechanism involving: (a) direct intestinal activation of PGC-1α-mediated mitochondrial fatty acid oxidation and suppression of lipotoxic lipid intermediate accumulation; (b) indirect modulation of gut microbiota composition and its metabolome, leading to altered microbial metabolite signaling to the liver; and (c) attenuation of systemic oxidative stress and inflammatory chemokine cascades via NF-κB pathway regulation.

Collectively, this investigation bridges a significant evolutionary gap in our understanding of iminosugar bioactivity in non-mammalian vertebrates, positions sturgeon as an informative model for studying the evolutionary conservation of the GLA in lipid homeostasis, and provides a systems-level mechanistic framework for the use of natural metabolic modulators in mitigating diet-induced metabolic dysfunction.

## 2. Materials and Methods

### 2.1. Experimental Design

A basal diet was formulated based on the established nutritional requirements of white sturgeon (*Acipenser transmontanus*) [24] and previous studies on *A. schrenckii* [25]. The basal diet was then divided into four equal batches, to which purified 1-deoxynojirimycin (1-DNJ; purity ≥ 98.0%; CAS No. 19130-96-2; MedChemExpress, Monmouth Junction, NJ, USA) was added at graded inclusion levels of 0, 5, 10, and 15 mg/kg, respectively. Fishmeal was used as the primary dietary protein source, while fish oil and soybean oil served as the principal lipid sources, the full ingredient of all experimental diets are listed in Table S1. All diets were extruded into 3 mm sinking pellets using an EXT50A twin-screw feed extruder (Yanggong Machinery Co., Ltd., Beijing, China). The temperatures of the feeding, compression, and metering zones were set to 92 °C, 121 °C, and 87 °C, respectively. Pellets were air-dried at room temperature and stored at −20 °C until feeding.

### 2.2. Animal Management

A total of 600 juvenile *A. schrenckii* were obtained from the Beijing Tianyuanyugang Farm Co., Ltd. (Beijing Amur Sturgeon Elite Breeding Base, Beijing, China). The experimental fish were of undetermined sex, had not undergone any genetic modification, and had not received any prior experimental treatment before this trial. All fish were subjected to a 4-week acclimation period under standardized rearing conditions prior to the commencement of the feeding trial. Following a 24 h feed deprivation, 360 healthy fish with uniform initial body weight (IBW = 163.58 ± 0.36 g) were randomly selected and allocated to four dietary treatments, with three biological replicates per treatment. Each replicate was housed in an independent net cage containing 30 fish. During the 8-week feeding trial, fish were handed to apparent satiation twice daily, and uneaten feed was collected to determine feed intake. Water quality parameters were maintained within stable ranges: water temperature 20 ± 2 °C, pH 7.5–8.5, and dissolved oxygen ≥ 7.0 mg/L. Fish health was monitored daily; for individuals exhibiting severe stress, injury, or loss of appetite, pre-established humane endpoints were applied. All animal procedures were performed in accordance with the ARRIVE 2.0 guidelines for the care and use of experimental animals. All experiments were approved by the Inspection Form for the Guizhou University Experimental Animal Ethics (approval No. EAE-GZU-20240E072; approval date: 31 December 2024).

The overall survival rate was recorded daily throughout the trial and did not differ among the dietary groups (*p* > 0.05; Section 3.1 and Table S4). At the end of the 8-week feeding trial, fish were fasted for 24 h and anesthetized with tricaine methanesulfonate (MS-222; 100 mg/L) until loss of equilibrium. Blood was collected from the caudal vein using disposable blood collection needles and tubes. Blood samples for serum biochemistry were allowed to clot at 4 °C for 2 h and then centrifuged at 1000× *g* for 10 min at 4 °C. The serum was collected and stored at −80 °C until analysis. Intestinal tissues and contents were snap-frozen in liquid nitrogen and stored at −80 °C for intestinal microbiota analysis. A portion of the intestinal tissues was snap-frozen in liquid nitrogen and stored at −80 °C for untargeted metabolomics and transcriptomics analyses. Another portion was placed in RNAlater at 4 °C overnight and then stored at −80 °C for qPCR. For each dietary group, six fish (two randomly selected from each of the three replicate cages) were used for 16S rRNA sequencing, untargeted metabolomics, and transcriptome sequencing, whereas three fish (one per replicate cage) were used for qRT-PCR validation, so that sample numbers are aligned with the cage-level statistical design described above. Between-cage variance was retained in all analyses, and no individual-level data were used as independent replicates.

### 2.3. Growth Performance

A set of somatic and growth indicators was calculated to assess the growth status of Amur sturgeon, including weight gain rate (WGR), specific growth rate (SGR), feed conversion ratio (FCR), feed rate (FR), survival rate (SR), condition factor (CF), hepatosomatic index (HSI) and viscerosomatic index (VSI). All calculations were performed based on replicate-level data. The corresponding computational formulas are presented in Table S2. WGR (%) = 100 × (Wt − W_0_)/W_0_; SGR (%/day) = 100 × [ln(Wt) − ln(W_0_)]/days of feeding trial; FCR = feed intake/(Wt − W_0_ + weight of dead experimental fish); FR (%) = 100 × total dry matter intake of feed/[(Wt + W_0_)/2 × days of feeding trial]; SR (%) = 100 × terminal quantity/original quantity; CF = 100 × (body weight)/(body length)^3^; HSI (%) = 100 × (hepatic weight)/(body weight); VSI (%) = 100 × (viscus weight)/(body weight), where W_0_ and Wt are the initial and final body weights (g), respectively.

### 2.4. Serum Biochemistry

Total cholesterol (TC; cat. No. A111-1-1), triglyceride (TG; cat. No. A110-1-1), glucose (GLU; cat. No. A154-1-1), total antioxidant capacity (T-AOC; cat. No. A015-2), superoxide dismutase (SOD; cat. No. A001-1), malondialdehyde (MDA; cat. No. A003-1), complement 3 (C3; cat. No. H186-1), complement 4 (C4; cat. No. H186-2), immunoglobulin M (IgM; cat. No. H109-1), total protein (TP; cat. No. A045-1-1), and alkaline phosphatase (ALP; cat. No. A059-2) assay kits were purchased from Nanjing Jiancheng Bioengineering Institute (Nanjing, China). All biochemical measurements were performed strictly according to the manufacturer-provided protocols.

### 2.5. 16S rRNA Gene Sequencing and Bioinformatics Analysis

Gut tissue and gut content samples were collected, immediately frozen in liquid nitrogen, and stored at −80 °C until DNA extraction. Total genomic DNA was extracted using the MagBeads FastDNA Kit (MP Biomedicals, CA, USA), and its concentration and purity were assessed via a NanoDrop NC2000 spectrophotometer (Thermo Fisher Scientific, Waltham, MA, USA) and 1% agarose gel electrophoresis. The V3–V4 hypervariable region of the bacterial 16S rRNA gene was amplified using the primer pair 338F and 806R. Amplicons were purified with VAHTS DNA Clean Beads (Vazyme, Nanjing, China), quantified using the Quant-iT PicoGreen dsDNA Assay Kit (Invitrogen, Carlsbad, CA, USA), pooled in equimolar concentrations, and subjected to paired-end sequencing (2 × 300 bp) on an Illumina NovaSeq 6000 platform (Personal Biotechnology Co., Ltd., Shanghai, China).

Raw sequencing reads were quality-filtered, denoised, and merged using the DADA2 plugin within QIIME 2 (v2023.5), and amplicon sequence variants (ASVs) were clustered into operational taxonomic units (OTUs) at 97% similarity. Alpha-diversity indices (Chao1, observed species, Shannon, and Simpson) were calculated to evaluate community richness and evenness. Beta-diversity was assessed using Bray–Curtis dissimilarity and visualized via non-metric multidimensional scaling (NMDS). Group-wise differences in microbial community structure were tested using permutational multivariate analysis of variance (PERMANOVA) and the permutational dispersion test (PermDisp), implemented in the R package vegan (v2.6-4). Linear discriminant analysis effect size (LEfSe) was applied to identify differentially abundant taxa between dietary groups, with significance set at *p* < 0.05 and a logarithmic LDA score threshold > 3. Microbial functional profiles were predicted using PICRUSt2 (v2.5.2) against the MetaCyc (v25.0) and KEGG (release 110.0) databases. All statistical analyses were performed in R v4.3.3.

### 2.6. Untargeted Metabolomics Analysis of Gut Tissues

Gut tissue samples (~30 mg) were homogenized in 200 μL of pre-chilled water using a high-throughput tissue grinder, followed by extraction with 800 μL of methanol:acetonitrile (1:1, *v*/*v*). The mixture was ultrasonicated for 30 min and incubated at −20 °C for 45 min to precipitate proteins. After centrifugation at 12,000 rpm (13,800× *g*) for 20 min at 4 °C, the supernatant (800 μL) was vacuum-dried. The residue was reconstituted in 150 μL of 50% aqueous methanol containing 5 ppm 2-chloro-L-phenylalanine (internal standard), vortexed, centrifuged, and filtered through a 0.22 μm PTFE membrane prior to UPLC-MS/MS analysis. A pooled QC sample was prepared by mixing equal aliquots of each sample filtrate and injected periodically to monitor instrument stability.

Chromatographic separation was performed on an ACQUITY UPLC HSS T3 column (1.8 μm, 2.1 mm × 100 mm) at 40 °C, with a flow rate of 0.4 mL/min. The mobile phases consisted of 0.1% formic acid in water (A) and 0.1% formic acid in acetonitrile (B), using a gradient elution program. The injection volume was 2 μL. Mass spectrometric detection was carried out on an Orbitrap Exploris 120 mass spectrometer (Thermo Fisher Scientific, MA, USA) equipped with a heated electrospray ionization (HESI) source, operating in both positive- and negative-ion modes. Full MS scans were acquired at a resolution of 60,000 (FWHM) over a mass range of *m*/*z* 70–1050, and data-dependent MS/MS fragmentation was performed at a resolution of 15,000 with stepped normalized collision energies of 20, 40, and 60 eV. Data acquisition was controlled by Xcalibur software (v4.7).

Raw data were processed using MS-DIAL (v4.9.221218) for peak detection, alignment, and metabolite annotation, with MS/MS spectra matched against HMDB (v5.0), MassBank (v2022.12), and METLIN (mass tolerance: 10 ppm for precursor ions, 20 ppm for fragment ions; similarity score > 0.7). Given that these databases are primarily human/mammalian-focused, metabolite identifications in sturgeon should be considered putative. However, KEGG pathway annotations are available for sterlet (*Acipenser ruthenus*), and pathway enrichment analysis was performed against these species-level annotations, providing a sturgeon-specific reference for pathway-level interpretation [citation:KEGG sterlet]. Features with >50% missing values across all samples or a relative standard deviation (RSD) > 30% in QC samples were excluded. The remaining data matrix was subjected to missing value imputation (half of the minimum value) and Pareto scaling normalization. Principal component analysis (PCA) and orthogonal partial least-squares discriminant analysis (OPLS-DA) were performed using the R package ropls (v1.28.0). Differential metabolites were identified based on variable importance in projection (VIP) > 1.0, fold change (FC) > 1, and *p* < 0.05. Kyoto Encyclopedia of Genes and Genomes (KEGG) pathway enrichment analysis was conducted using the R package clusterProfiler (v4.6.0) with sterlet (*A. ruthenus*) pathway annotations. This multi-dimensional metabolomic profiling was designed to capture the gut tissue metabolic responses to DNJ supplementation, providing a molecular basis for subsequent integrative analyses with gut microbial and transcriptomic datasets.

### 2.7. Transcriptome Sequencing and Analysis

Gut tissue samples were collected, snap-frozen in liquid nitrogen, and stored at −80 °C until RNA extraction. Total RNA was isolated using TRIzol reagent (Invitrogen, Carlsbad, CA, USA). RNA concentration and purity were assessed with a NanoDrop One spectrophotometer (Thermo Fisher Scientific, MA, USA), and RNA integrity was evaluated using agarose gel electrophoresis and the Agilent Fragment Analyzer 5400 system (Agilent Technologies, Santa Clara, CA, USA). High-quality RNA samples (RIN > 7.0) were subjected to mRNA enrichment using oligo(dT) magnetic beads. The enriched mRNA was fragmented and reverse-transcribed into cDNA, followed by end repair, A-tailing, adapter ligation, and PCR amplification to construct cDNA libraries. The libraries were sequenced on the Illumina NovaSeq X Plus platform (Illumina, Santa Clara, CA, USA) with a paired-end 150 bp read length.

Raw sequencing reads were processed with Fastp (v0.22.0) for quality filtering (removing adapters and low-quality reads) to generate clean reads. Clean reads were aligned to the reference genome using HISAT2 (v2.1.0). Gene expression levels were quantified as fragments per kilobase of transcript per million mapped reads (FPKM). Differential expression analysis was performed using DESeq2 (v1.38.3). Genes meeting |log_2_ fold change| ≥ 1 and Benjamini–Hochberg adjusted *p* < 0.05 (FDR < 0.05) were defined as differentially expressed genes (DEGs). Because the FDR-controlled DEG set was limited in size, an additional exploratory DEG set was defined using |log_2_ fold change| ≥ 1 and raw *p* < 0.05, and this set was used for GO and KEGG enrichment analyses as explicitly labeled exploratory analyses. Expression patterns of DEGs were visualized via heatmaps generated with the ComplexHeatmap package (v2.16.0). Gene Ontology (GO) functional annotation and Kyoto Encyclopedia of Genes and Genomes (KEGG) pathway enrichment analyses were implemented using clusterProfiler (v4.6.0), with significantly enriched terms identified at *p* < 0.05.

### 2.8. Quantitative Real-Time PCR (qRT-PCR) Validation

Total RNA was extracted from the same gut tissue samples using the RNAsimple Total RNA Kit (Cat. No. DP419, Tiangen Biotech, Beijing, China). First-strand complementary DNA (cDNA) was synthesized from 1 μg of high-quality total RNA (A260/A280 > 1.8) using the PrimeScript RT reagent Kit with gDNA Eraser (Cat. No. RR047A, Takara Biotechnology Inc., Shiga, Japan). qRT-PCR was performed in a 20 μL reaction volume containing gene-specific qPCR primers, SYBR Green Premix Ex Taq II (Takara, Cat. No. RR820A), and cDNA template, following the manufacturer’s protocol. The thermal cycling conditions were as follows: initial denaturation at 95 °C for 30 s, followed by 40 cycles of 95 °C for 5 s and 60 °C for 34 s. Melting curve analysis was conducted to verify the specificity of each amplification. The β-actin gene (GenBank accession No. AY649619) was used as the internal reference for normalization. Relative gene expression levels were calculated using the 2^−ΔΔCt^ method. All reactions were performed with three biological replicates (*n* = 3 cages per treatment, one fish per cage). To exclude pseudo-replication, the three biological replicates were drawn from three different replicate cages, so that the replicates were statistically independent. The primer sequences for all target and reference genes are listed in Table S3.

### 2.9. Correlation Analysis of Multi-Omics Data

Pearson correlation analysis was further performed to evaluate the regulatory relationships between gene co-expression modules and metabolite modules. Module–module pairs with *p* < 0.05 were considered significantly correlated. The top 20 module–module pairs ranked by absolute correlation coefficient were visualized in a heatmap to characterize the overall transcriptome–metabolome interaction patterns.

### 2.10. Statistical Analysis

All statistical analyses were performed using GraphPad Prism (v10.6.0; San Diego, CA, USA). Normality and homogeneity of variance were assessed using the Shapiro–Wilk test and Levene’s test, respectively. Growth performance and serum biochemical parameters were analyzed by one-way ANOVA, while qRT-PCR expression data were analyzed by two-way ANOVA with dose and gene as fixed factors, followed by Tukey’s HSD post hoc test for pairwise comparisons. Data are presented as mean ± SEM (*n* = 6 per group). Statistical significance was set at *p* < 0.05. Growth performance and serum biochemical parameters are expressed at the level of the replicate cage (*n* = 3 cages per group), consistent with the cage-level experimental unit defined in Section 2.2. Gut microbiota (16S rRNA), untargeted metabolomic, and transcriptomic datasets are expressed as *n* = 6 per group (two fish per cage, three cages per group), and qRT-PCR data as *n* = 3 per group (one fish per cage, three cages per group).

## 3. Results

### 3.1. Growth Performance and Morphological Parameters

No significant differences in final body weight (FBW), survival rate (SR), weight gain rate (WGR), specific growth rate (SGR), feeding rate (FR), or condition factor (CF) were observed among all groups (*p* > 0.05). However, the hepatosomatic index (HSI) and viscerosomatic index (VSI) were significantly increased in the DNJ10 group, and the feed conversion ratio (FCR) was significantly elevated in the DNJ15 group (*p* < 0.05). Detailed growth and morphological data are provided in Table S4.

### 3.2. Serum Biochemical Parameters

**Figure 1:**
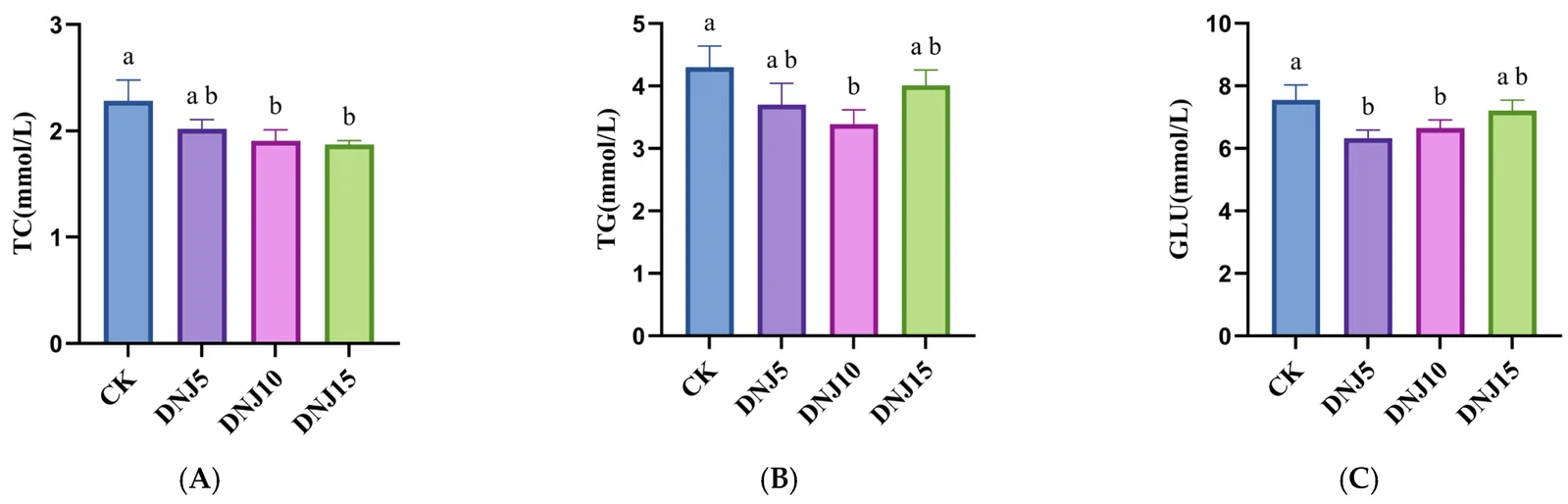

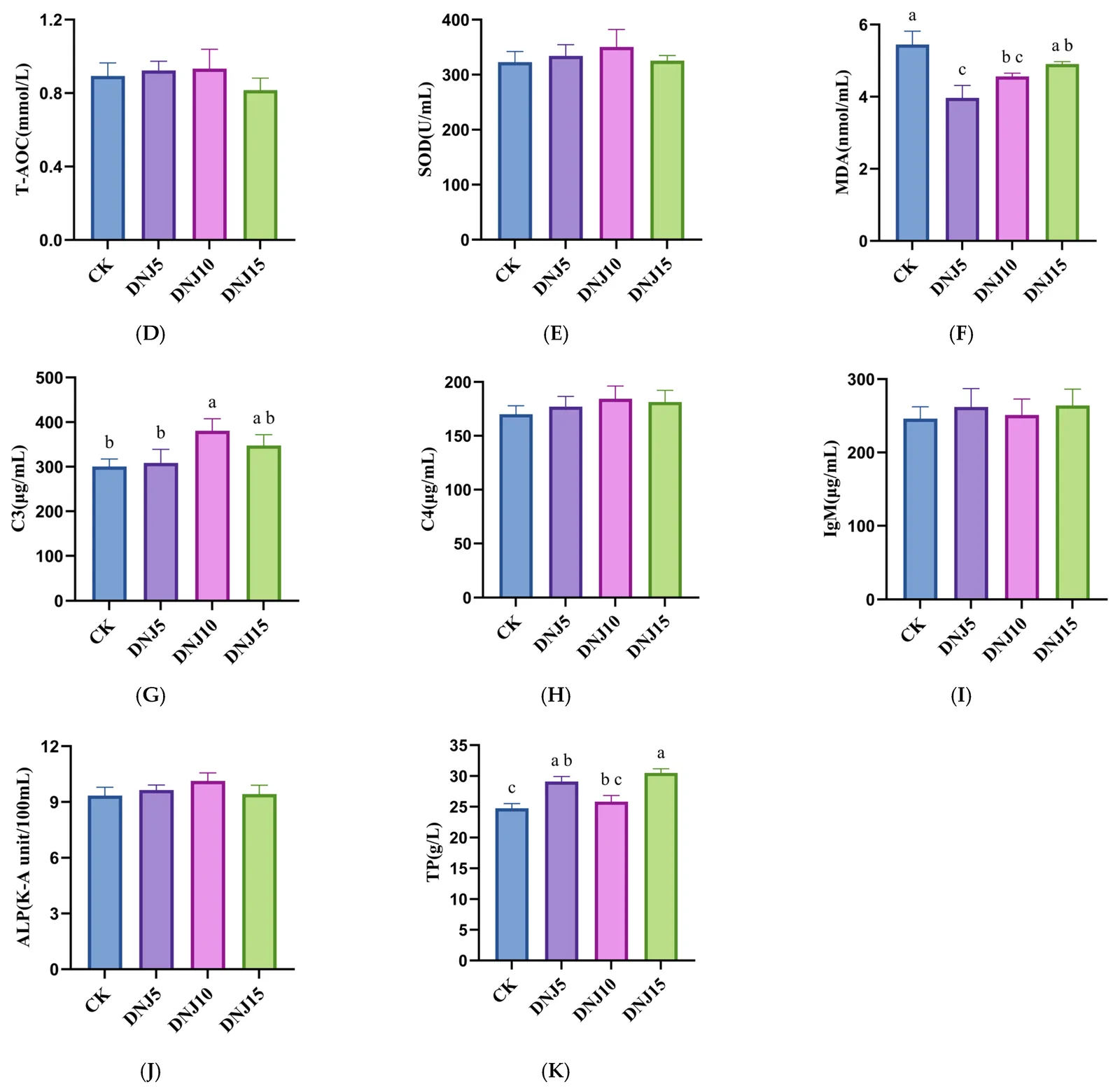
Serum biochemical parameters of juvenile *A. schrenckii* fed diets supplemented with graded levels of DNJ (*n* = 3). Bars with different lowercase letters indicate significant differences among groups (*p* < 0.05). (**A**) Total cholesterol (TC); (**B**) triglyceride (TG); (**C**) glucose (GLU); (**D**) total antioxidant capacity (T-AOC); (**E**) superoxide dismutase (SOD); (**F**) malondialdehyde (MDA); (**G**) complement component 3 (C3); (**H**) complement component 4 (C4); (**I**) immunoglobulin M (IgM); (**J**) alkaline phosphatase (ALP); (**K**) total protein (TP).

Dietary DNJ supplementation differentially modulated serum biochemical parameters (Figure 1). Serum TG was significantly reduced in the DNJ10 group (*p* < 0.05), while TC was significantly decreased in both the DNJ10 and DNJ15 groups relative to the control (*p* < 0.05). Serum GLU levels were markedly lower in the DNJ5 and DNJ10 groups (*p* < 0.05). Quadratic regression analysis indicated that the theoretical minimum TG and GLU concentrations corresponded to dietary DNJ levels of 8.48 and 7.89 mg/kg, respectively (*p*_quadratic_ < 0.05). Regarding oxidative stress markers, MDA content was significantly decreased in the DNJ5 and DNJ10 groups (*p* < 0.05), with the theoretical minimum MDA corresponding to a dietary DNJ level of 8.06 mg/kg (*p*_quadratic_ < 0.05). TP was significantly elevated in the DNJ5 and DNJ15 groups, and C3 was significantly increased in the DNJ10 group (*p* < 0.05). Although T-AOC, SOD, C4, IgM, and ALP exhibited increasing trends with DNJ supplementation, these changes did not reach statistical significance (*p* > 0.05). Collectively, these results indicate that DNJ supplementation exerts dose-dependent beneficial effects on serum lipid profiles, glucose homeostasis, oxidative status, and immune parameters in sturgeon.

### 3.3. Intestinal Microbial Diversity and Community Composition

#### 3.3.1. Alpha and Beta Diversity of Gut Microbiota

**Figure 2:**
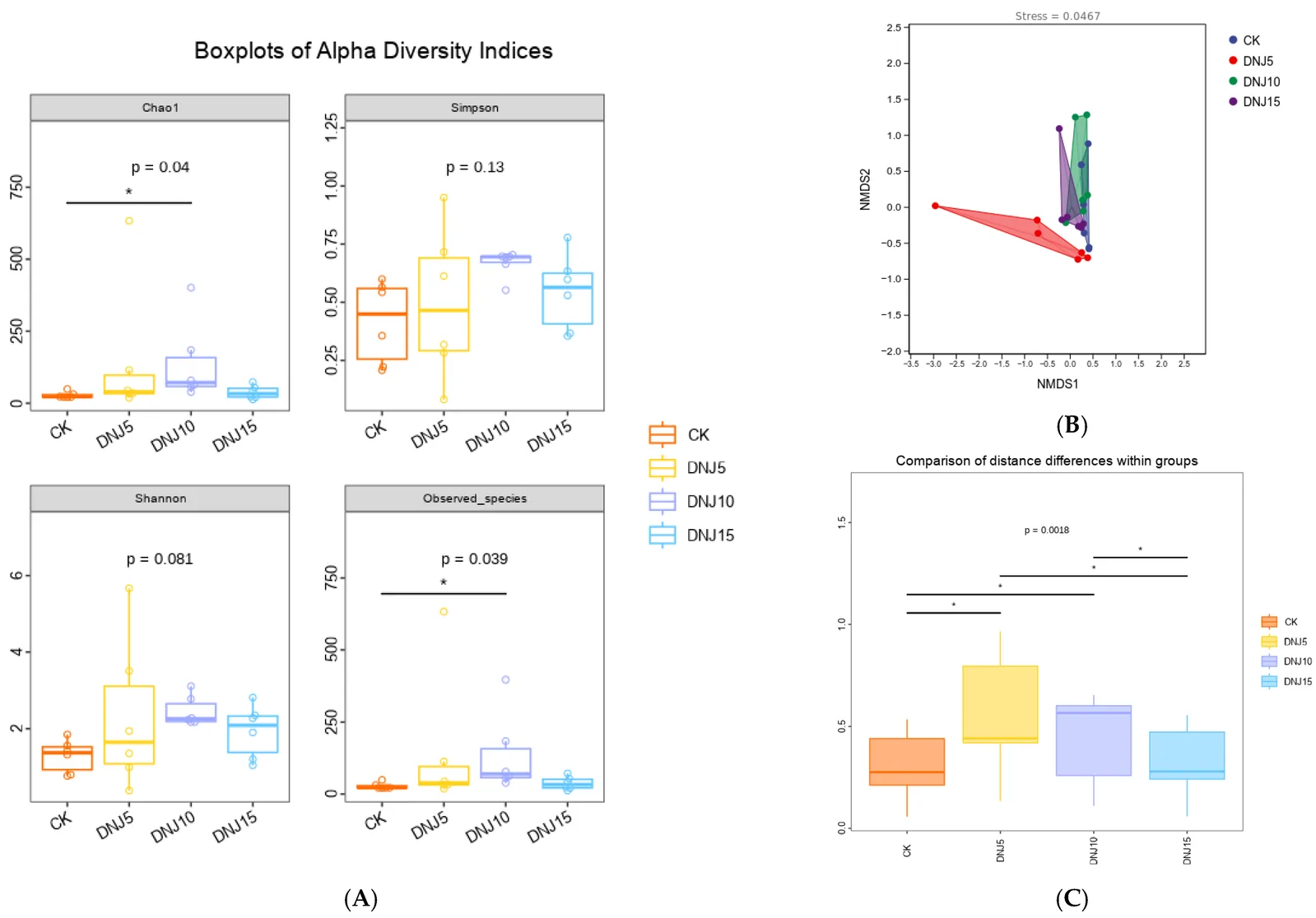
Effects of dietary DNJ supplementation on intestinal microbial diversity in sturgeon (*n* = 6). (**A**) Alpha-diversity indices (Chao1, observed species, Shannon, and Simpson) of gut microbiota among experimental groups. (**B**) Beta-diversity visualized by NMDS analysis based on Bray–Curtis distance. (**C**) Boxplot of Bray–Curtis distance dissimilarity among groups, with PERMANOVA results indicating significant overall differences in microbial community structure (*p* = 0.0018). Asterisks indicate significant differences (* *p* < 0.05).

Compared with the control group, the Chao1 index and observed species in the DNJ10 group were significantly higher than those in the control group (*p* < 0.05), while no significant intergroup differences were found in the Shannon and Simpson indices (*p* > 0.05; Figure 2A).

NMDS analysis was performed based on the Bray–Curtis distance matrix. The ordination stress value was 0.0467 (<0.05), indicating that the two-dimensional NMDS ordination adequately captured the β-diversity structure among groups (Figure 2B). The PERMDISP test confirmed the homogeneity of dispersion across all groups (*p* > 0.05), satisfying the prerequisite assumption for PERMANOVA. PERMANOVA revealed a significant difference in the overall structure of gut microbiota among dietary treatment groups (*p* = 0.0018; Figure 2C).

#### 3.3.2. Taxonomic Composition at the Phylum and Genus Levels

**Figure 3:**
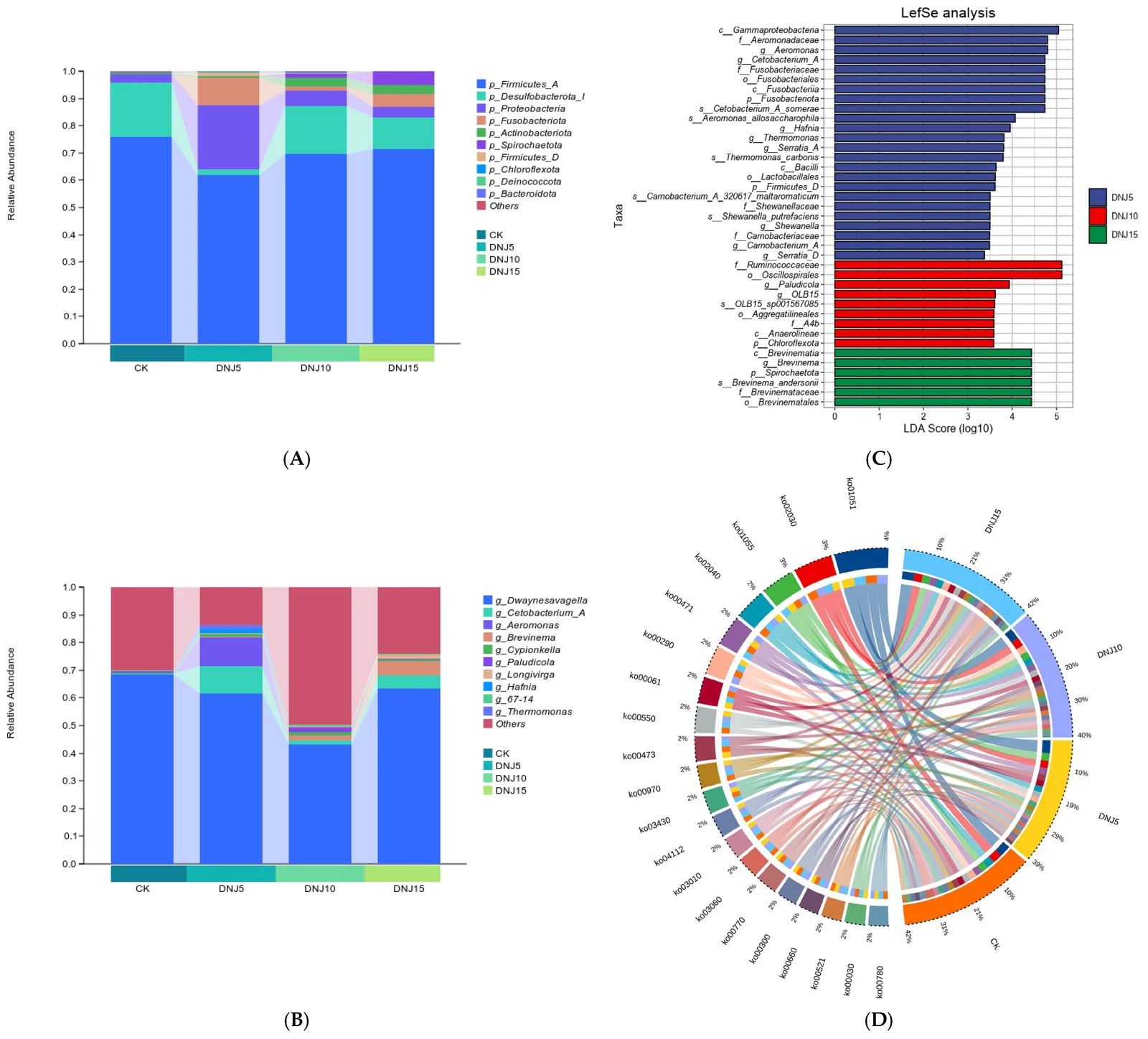
Effects of dietary DNJ supplementation on gut microbial community composition and functional profiles (*n* = 6). (**A**) Relative abundance of microbial communities at the phylum level. (**B**) Relative abundance at the genus level. (**C**) Taxonomic biomarkers of differential gut microbiota among groups identified by LEfSe, with an LDA score threshold > 3 and *p* < 0.05. (**D**) Circos diagram illustrating the distribution of KEGG functional pathway abundances in gut microbiota across dietary groups. The inner rings represent functional units (KEGG pathways) or sample groups, and the outer rings display the relative abundance composition of each functional unit or sample.

Dietary DNJ supplementation induced marked shifts in the intestinal microbial community composition at both the phylum and genus levels (Figure 3A,B). At the phylum level, the control group (CK) was overwhelmingly dominated by Firmicutes_A (75.9%), followed by Desulfobacterota (19.8%). In the DNJ5 group, the relative abundance of Firmicutes_A decreased, whereas Proteobacteria increased and became the second dominant phylum; the abundance of Fusobacteriota was also elevated relative to CK. In the DNJ10 group, Firmicutes_A remained the dominant phylum, accompanied by the recovery of Desulfobacterota, and Fusobacteriota abundance was moderately increased. Similarly, Firmicutes_A predominated in the DNJ15 group with restored Desulfobacterota, while Fusobacteriota abundance remained higher than in CK.

At the genus level, the CK group was overwhelmingly dominated by unclassified *Dwaynesavagella*, which accounted for 68.53% of the total community, whereas the abundances of other genera were extremely low, indicating a structurally simple microbial community dominated by a single genus. In the DNJ5 group, besides *Dwaynesavagella*, the relative abundances of *Cetobacterium_A*, and *Aeromonas* were markedly increased, resulting in noticeable improvement in microbial diversity. The overall microbial structure of the DNJ10 group was similar to that of CK; however, specific genera including *Cetobacterium_A* and *Paludicola* were distinctly enriched, suggesting a moderate compositional shift following DNJ supplementation. In the DNJ15 group, in addition to the dominant *Dwaynesavagella*, *Cetobacterium_A* and *Brevinema* emerged as newly enriched predominant genera, further reshaping the intestinal microbial structure.

#### 3.3.3. Differential Microbial Taxa Identified by LEfSe Analysis

LEfSe analysis identified distinct taxonomic signatures associated with each dietary DNJ regimen (LDA > 3, *p* < 0.05; Figure 3C). The signature microbiota of the DNJ5 group were mainly derived from the class Gammaproteobacteria and the phylum Fusobacteriota, with *Cetobacterium somerae* and the genus *Aeromonas* as the core differential taxa. The biomarker taxa of the DNJ10 group were primarily affiliated with Firmicutes_A and Chloroflexota, represented by the family Ruminococcaceae and the genus *Paludicola*. The differential biomarkers in the DNJ15 group were predominantly derived from the phylum Spirochaetota, dominated by *Brevinema andersonii* and its affiliated taxa.

#### 3.3.4. Functional Prediction of Gut Microbiota (PICRUSt2)

PICRUSt2-based functional prediction revealed distinct KEGG pathway enrichment patterns among dietary groups (Figure 3D). Pathways associated with bacterial environmental sensing, colonization, and virulence—including bacterial chemotaxis (ko02030), flagellar assembly (ko02040), quorum sensing (ko01055), and the two-component system (ko01051)—were predominantly derived from the CK group. In contrast, pathways related to nutritional metabolism, such as fatty acid biosynthesis (ko00061), valine, leucine, and isoleucine biosynthesis (ko00290), and D-glutamine and D-glutamate metabolism (ko00471), were mainly enriched in the DNJ5 group. The DNJ10 group was characterized by the enrichment of pathways involved in genetic information processing, DNA repair, and fundamental carbohydrate metabolism, including ribosome (ko03010), protein export (ko03060), mismatch repair (ko03430), pantothenate and CoA biosynthesis (ko00770), and the pentose phosphate pathway (ko00300). The DNJ15 group exhibited a relatively balanced distribution across diverse metabolic pathways, with no single functional category dominating, suggesting a more functionally diversified microbial community. Core housekeeping pathways, including peptidoglycan biosynthesis (ko00550), D-alanine metabolism (ko00473), and aminoacyl-tRNA biosynthesis (ko00970), were detected in all four groups, representing the conserved core functions of the gut microbiota across all treatments. The Circos diagram comprehensively illustrated the relative abundance distribution and group-specific contributions of these functional pathways.

### 3.4. Untargeted Metabolomics of Gut Tissues

**Figure 4:**
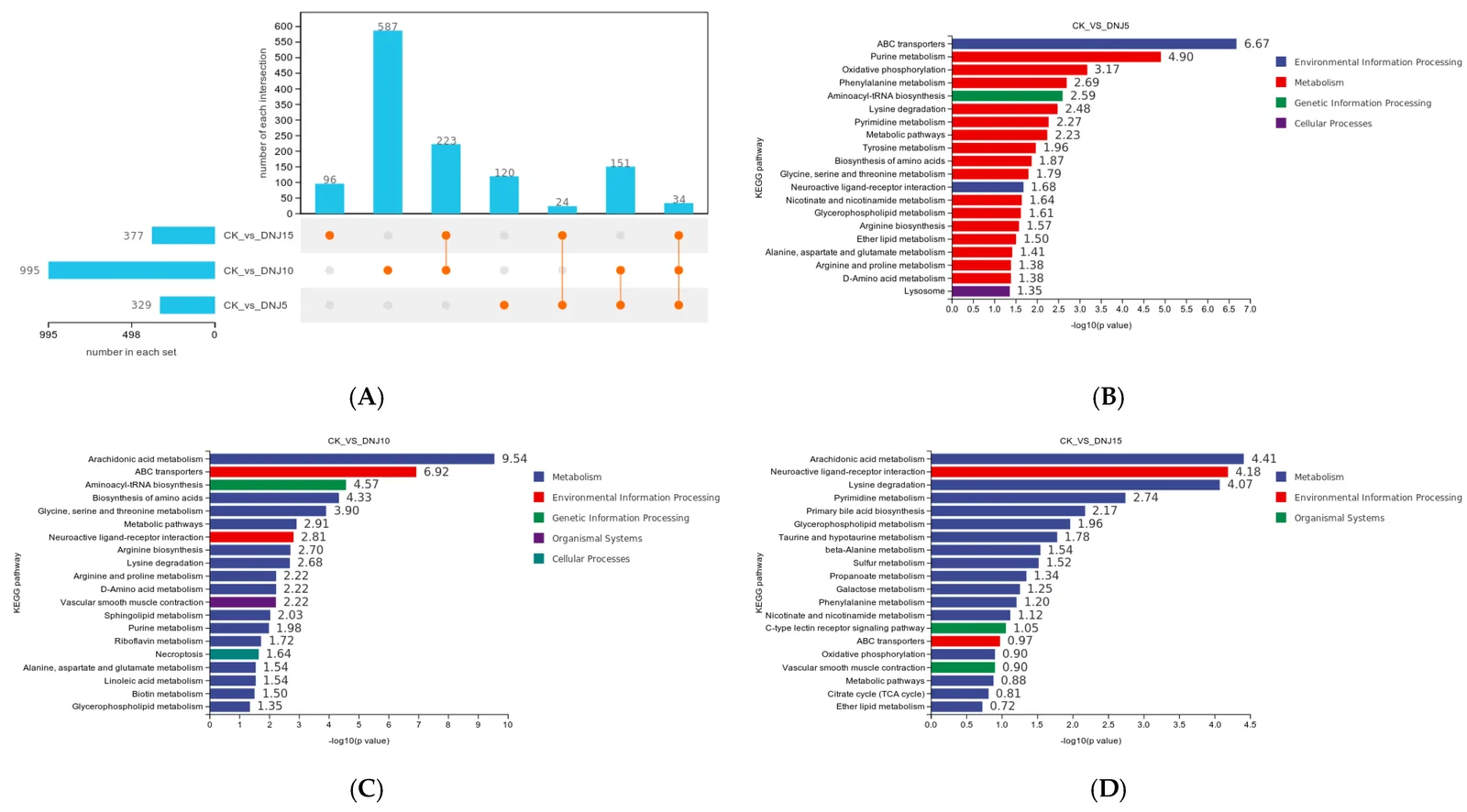
Differential metabolite distribution and KEGG pathway enrichment analysis (*n* = 6). (**A**) UpSet plot showing the distribution of shared and unique differential metabolites among comparison groups. (**B**–**D**) KEGG pathway enrichment analysis of differential metabolites derived from the comparisons of CK vs. DNJ5 (**B**), CK vs. DNJ10 (**C**), and CK vs. DNJ15 (**D**), respectively. The *x*-axis represents-− log10 (*p*-value) and the *y*-axis represents KEGG Pathway.

Untargeted metabolomic profiling of gut tissues was performed to evaluate the host metabolic response to dietary DNJ supplementation. Compared with the control group (DNJ0), a total of 329, 995, and 377 differentially accumulated metabolites were identified in the DNJ5, DNJ10, and DNJ15 groups, respectively, with the DNJ10 group exhibiting the most pronounced metabolic perturbation and a non-monotonic dose–response pattern. UpSet analysis revealed 34 core differential metabolites common to all three treatment groups. These shared metabolites were predominantly lipids and lipid-like molecules (38.2%), followed by organoheterocyclic compounds, benzenoids, organic acids and derivatives, and nucleosides, nucleotides, and analogues. Among these 34 core metabolites, 14 were consistently downregulated, 10 were consistently upregulated, 7 were upregulated in DNJ5 but downregulated in both DNJ10 and DNJ15, and the remaining 3 exhibited the opposite pattern (downregulated in DNJ5 but upregulated in DNJ10 and DNJ15), indicating dose-dependent shifts in the direction of metabolic regulation (Figure 4A).

KEGG pathway enrichment analysis of the differentially accumulated metabolites further revealed a gradient metabolic response under different DNJ supplementation doses. Metabolic perturbation in the low-dose group (DNJ5) was dominated by substance transport (ABC transporters), energy metabolism (oxidative phosphorylation), and basal amino acid metabolism (Figure 4B). The medium-dose group (DNJ10) was characterized by lipid metabolism (arachidonic acid metabolism) and amino acid biosynthesis and transport, with the highest number of significantly enriched pathways (*p* < 0.05) among all treatment groups, indicating the maximal magnitude of metabolic perturbation (Figure 4C). Although lipid metabolism remained responsive in the high-dose group (DNJ15), neuroactive ligand–receptor interaction and bile acid metabolism emerged as new regulatory foci, accompanied by a reduction in overall metabolic perturbation intensity (Figure 4D).

The ABC transporter pathway was among the two most significantly enriched pathways in both the DNJ5 and DNJ10 groups, serving as a core regulatory mechanism across low and medium doses (Figure 4B,C). Arachidonic acid metabolism was the most significantly enriched pathway in both the DNJ10 and DNJ15 groups, representing the key lipid metabolic response shared by medium and high doses (Figure 4C,D). The lysine degradation pathway was significantly enriched in all three treatment groups, acting as a dose-independent core amino acid metabolic response (Figure 4B–D).

### 3.5. Transcriptomic Analysis

RNA-seq data quality was high across all samples, with Q20 and Q30 values averaging 99.49% and 97.97%, respectively, ensuring sufficient sequencing depth for downstream analysis.

#### 3.5.1. Differentially Expressed Genes

**Figure 5:**
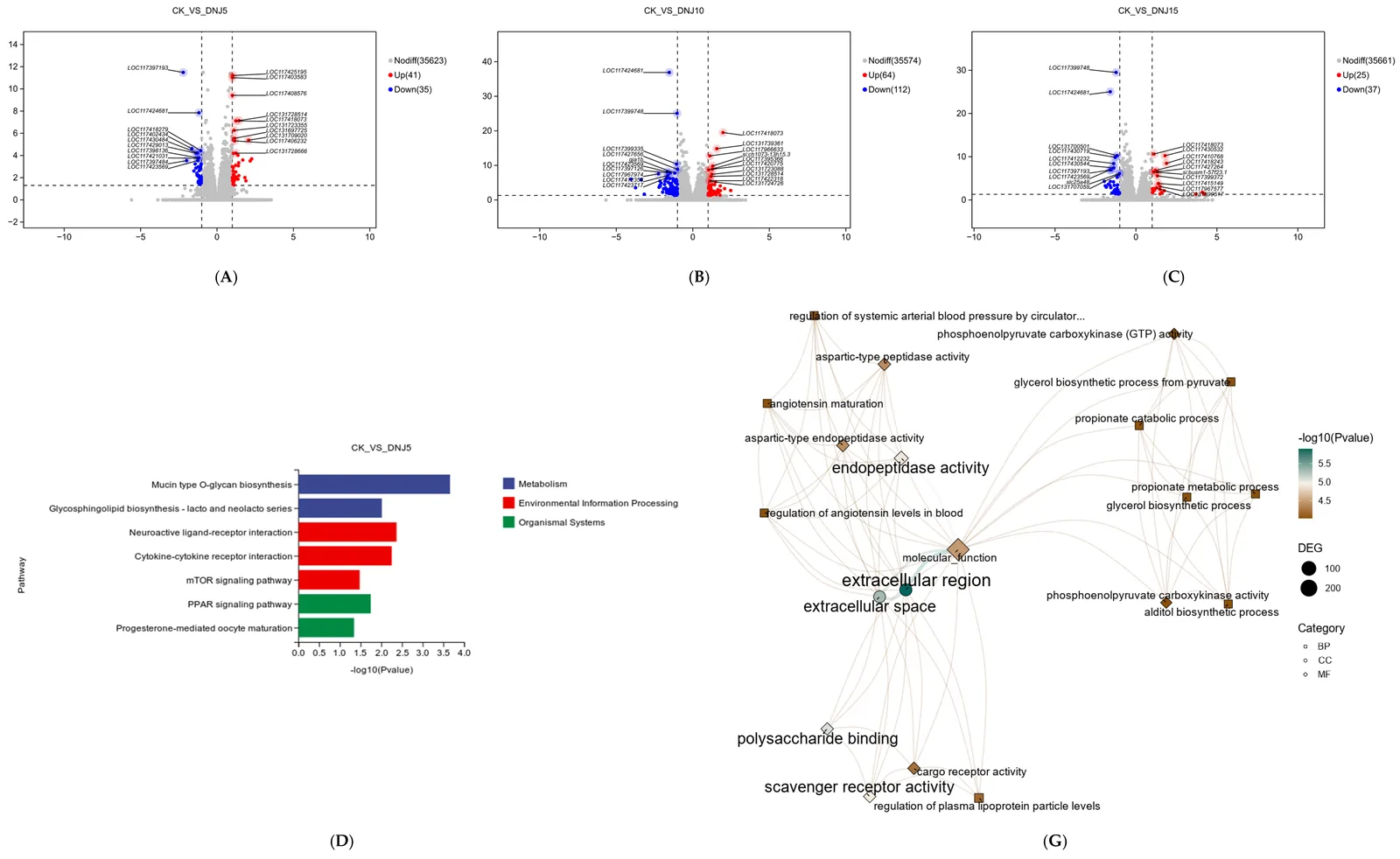

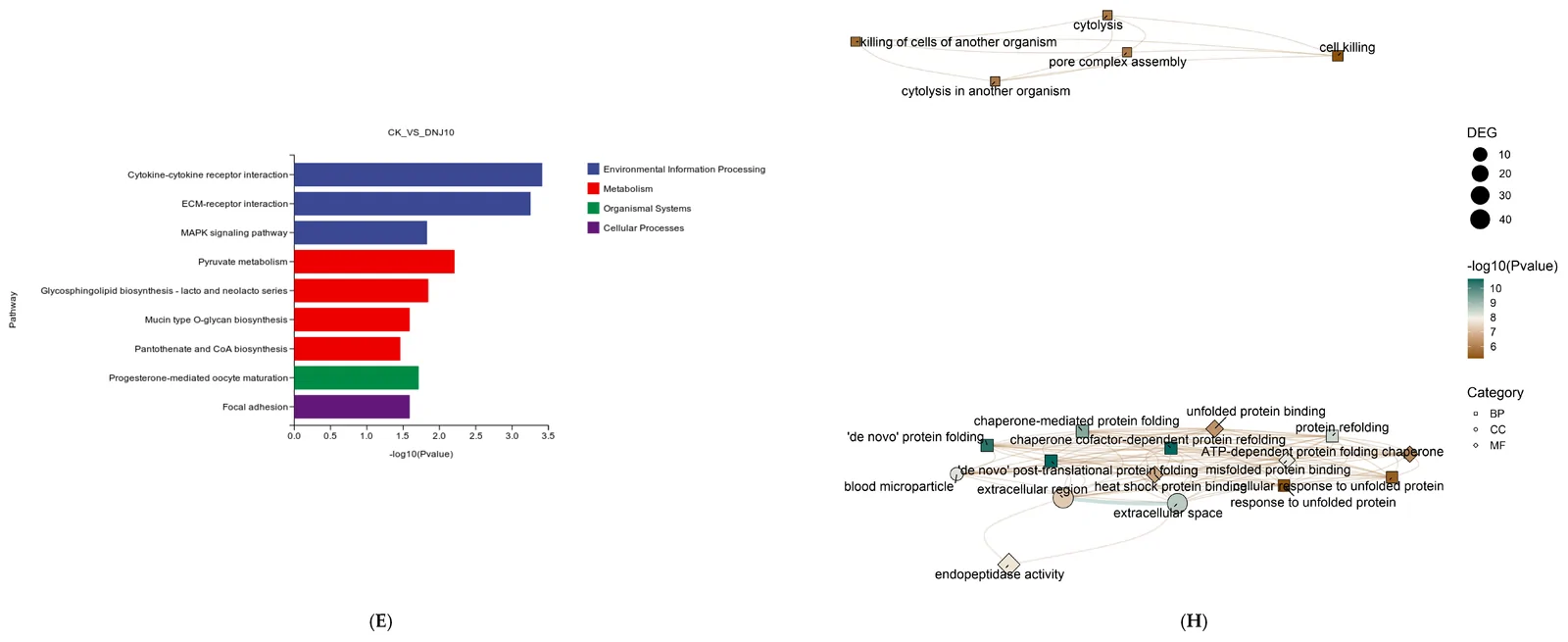

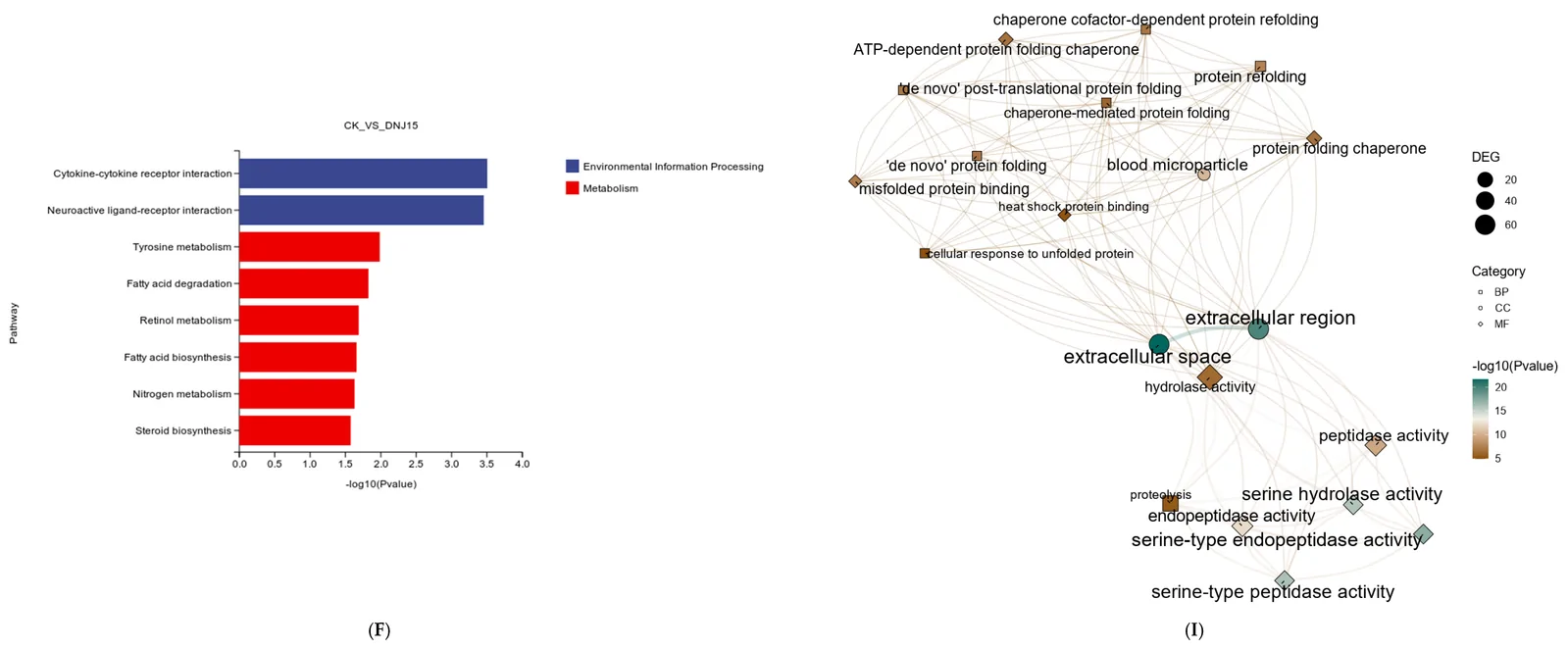
Differential gene expression and functional enrichment analyses across the three treatment comparisons (*n* = 6). (**A**–**C**) Volcano plots of the FDR-controlled DEG set (FDR < 0.05) for CK vs. DNJ5 (**A**), CK vs. DNJ10 (**B**), and CK vs. DNJ15 (**C**). Red and blue dots denote significantly up- and down-regulated genes, respectively; gray dots indicate non-significant genes. (**D**–**F**) KEGG pathway enrichment bar charts based on the exploratory DEG set (raw *p* < 0.05) for CK vs. DNJ5 (**D**), CK vs. DNJ10 (**E**), and CK vs. DNJ15 (**F**). The vertical axis lists the enriched KEGG pathways, and the horizontal axis represents −log_10_(*p*-value) of pathway enrichment. (**G**–**I**) GO enrichment circle plots based on the exploratory DEG set (raw *p* < 0.05) for CK vs. DNJ5 (**G**), CK vs. DNJ10 (**H**), and CK vs. DNJ15 (**I**). Each circle represents a GO term; circle size is proportional to the number of enriched DEGs within that term, and the color gradient reflects enrichment significance. The thickness of connecting lines represents the number of shared DEGs between paired GO terms, with thicker lines indicating greater overlap.

Compared with the CK group, 76 DEGs were identified at FDR < 0.05 in the DNJ5 group, including 41 up-regulated and 35 down-regulated genes. The DNJ10 group exhibited the highest number of DEGs at FDR < 0.05, with a total of 176 DEGs (64 up-regulated and 112 down-regulated). In the DNJ15 group, 62 DEGs were identified at FDR < 0.05, among which 25 were up-regulated and 37 were down-regulated (Figure 5A–C). Because the FDR-controlled DEG set was limited in size, GO and KEGG enrichment analyses were additionally performed on an exploratory DEG set defined by raw *p* < 0.05 (DNJ5: 405 DEGs; DNJ10: 498 DEGs; DNJ15: 384 DEGs), and these enrichment results are explicitly labeled as exploratory.

#### 3.5.2. KEGG Pathway Enrichment

KEGG pathway enrichment analysis of the exploratory DEG set (raw *p* < 0.05) revealed distinct functional profiles across the three DNJ supplementation groups. In the DNJ5 group, DEGs were mainly involved in metabolic pathways including carbohydrate metabolism and sphingolipid metabolism (ko00600), as well as environmental information-processing pathways such as neuroactive signal transduction, cytokine signaling, and the mTOR signaling pathway (Figure 5D). In the DNJ10 group, DEGs were significantly enriched in environmental information-processing pathways, including cytokine–receptor interaction, ECM–receptor interaction, and the MAPK signaling pathway, alongside metabolic pathways such as pyruvate metabolism and glycosphingolipid biosynthesis (Figure 5E). For the DNJ15 group, cytokine–receptor interaction and neuroactive ligand–receptor interaction remained significantly enriched, and additional metabolic pathways—including fatty acid metabolism and tyrosine metabolism (ko00350)—were broadly enriched at the highest DNJ dose (Figure 5F).

#### 3.5.3. GO Functional Enrichment

GO enrichment analysis of the exploratory DEG set (raw *p* < 0.05) was performed across the three DNJ supplementation groups, revealing a dose-dependent functional transition from metabolic processes at the low dose to protein homeostasis-related pathways at medium and high doses (Figure 5G–I). At the cellular component (CC) level, extracellular region and extracellular space were the most significantly enriched terms across all three groups, with the highest enrichment observed in the DNJ15 group. Blood microparticle was also significantly enriched in the DNJ10 and DNJ15 groups (Figure 5G–I).

In terms of molecular function (MF), the DNJ5 group exhibited significant enrichment of endopeptidase activity, polysaccharide binding, and scavenger receptor activity—functionally associated with extracellular matrix remodeling and innate immune recognition (Figure 5G). The DNJ10 group-maintained endopeptidase activity enrichment while additionally displaying significant enrichment of ATP-dependent protein folding chaperone and unfolded protein binding (Figure 5H). In the DNJ15 group, the MF profile shifted toward serine-type peptidase activity, hydrolase activity, and misfolded protein binding, indicating an enhanced capacity for proteolytic activity and protein quality control (Figure 5I).

For biological process (BP), DNJ5 was predominantly associated with metabolic and physiological regulatory processes, including glycerol biosynthetic process, propionate metabolic process, and angiotensin maturation (Figure 5G). In contrast, the DNJ10 and DNJ15 groups exhibited robust enrichment of protein folding-related processes, including chaperone-mediated protein folding, unfolded protein binding, response to unfolded protein, cellular response to unfolded protein, and protein refolding, with enrichment peaking in the DNJ15 group (Figure 5H,I). Collectively, these GO results indicate that DNJ supplementation at medium to high doses imposes substantial proteostatic pressure, while low-dose DNJ primarily activates metabolic and innate immune regulatory pathways.

### 3.6. Integrated Analysis of Transcriptome and Metabolome

**Figure 6:**
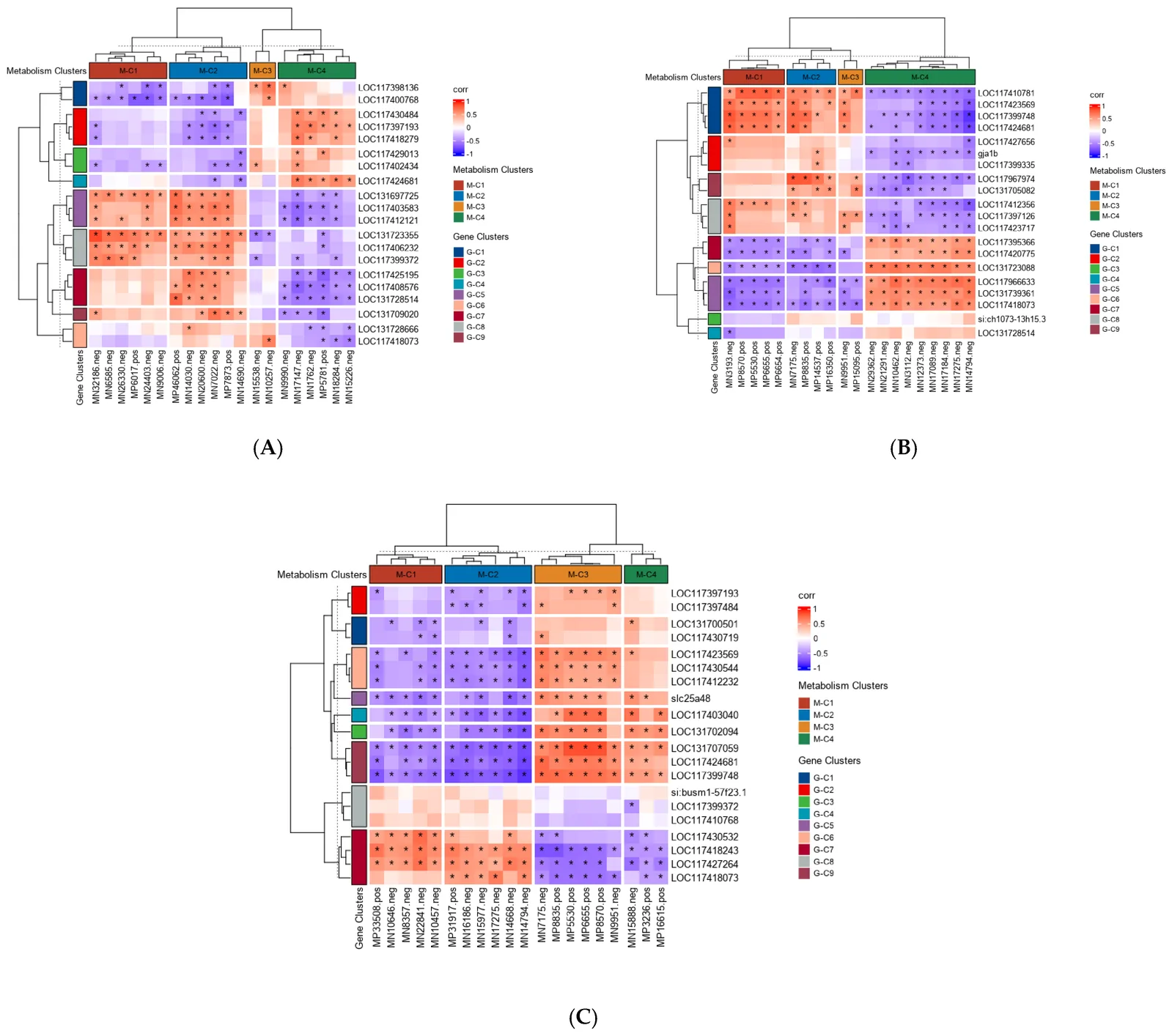
Pearson correlation heatmaps of genes and differential metabolites in the three treatment comparisons (*n* = 6). (**A**) DNJ5 vs. CK; (**B**) DNJ10 vs. CK; (**C**) DNJ15 vs. CK. The horizontal axis represents metabolites (grouped into metabolic modules M-C1 to M-C4), and the vertical axis represents genes (grouped into gene modules G-C1 to G-C9). Red indicates positive correlations, and blue indicates negative correlations, with color intensity reflecting the magnitude of the correlation coefficient. Asterisks indicate statistically significant correlations (*p* < 0.05). The top and left color bars indicate metabolite module and gene module assignments, respectively.

To explore the functional interactions between host transcriptional and metabolic responses, DEGs were clustered into nine co-expression modules (G-C1 to G-C9), and differentially accumulated metabolites were grouped into four metabolic modules (M-C1 to M-C4). Pearson correlation analysis was then performed between individual genes and metabolites, with module assignments indicated by color bars (Figure 6).

In the DNJ5 group, genes assigned to modules G-C5 and G-C8 exhibited significant positive correlations with metabolites in modules M-C1 and M-C2, while showing negative correlations with metabolites in M-C4. Genes assigned to G-C7 were positively correlated with metabolites in M-C2 and negatively correlated with those in M-C4. In contrast, genes assigned to G-C2 and G-C4 displayed significant positive correlations with metabolites in M-C4 (Figure 6A).

In the DNJ10 group, the correlation pattern underwent a marked shift: genes assigned to G-C1 showed positive correlations with metabolites in M-C1, M-C2, and M-C3, but negative correlations with those in M-C4. Conversely, genes assigned to G-C5, G-C6, and G-C7 exhibited negative correlations with metabolites in M-C1–M-C3 and positive correlations with those in M-C4 (Figure 6B). This reversed correlation pattern suggests a dose-dependent functional reorientation at the medium DNJ dose.

In the DNJ15 group, genes assigned to G-C3, G-C4, G-C5, G-C6, and G-C9 were negatively correlated with metabolites in M-C1 and M-C2, but positively correlated with those in M-C3 and M-C4. Genes assigned to G-C7 showed the opposite pattern, being positively correlated with metabolites in M-C1 and M-C2, and negatively correlated with those in M-C3 and M-C4 (Figure 6C). Collectively, these gene–metabolite correlations reveal a dose-dependent reconfiguration of transcriptome–metabolome interactions, with the DNJ10 group displaying the most distinct reversal of regulatory directions.

### 3.7. Integrated Transcriptomic and Metabolomic Analysis

**Figure 7:**
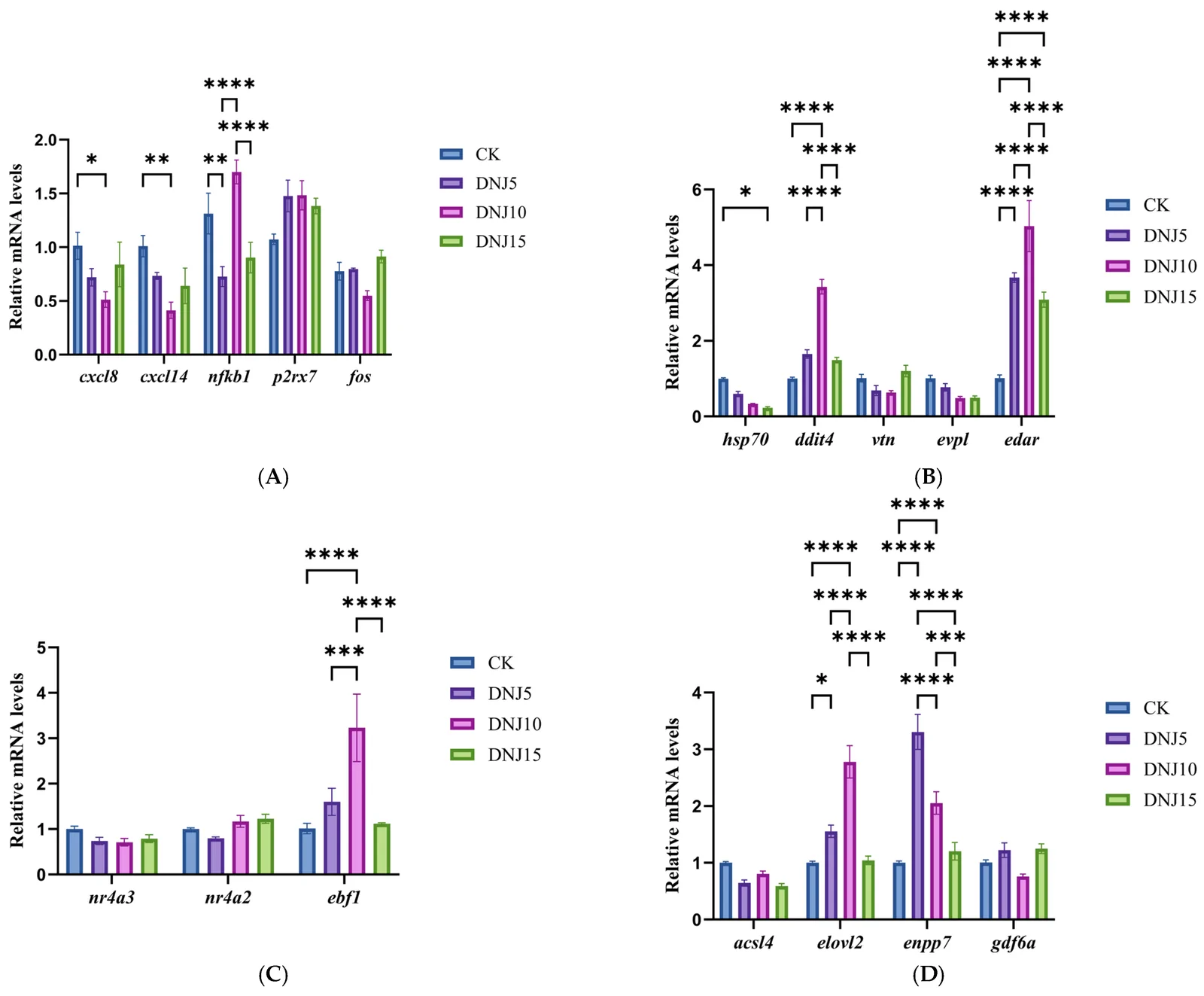
Effects of dietary DNJ supplementation on mRNA expression of functionally categorized genes in the gut (*n* = 3). (**A**) Inflammation and immune regulation: *cxcl8*, *cxcl14*, *nfkb1*, *p2rx7*, and *fos*. (**B**) Lipid metabolism and mitochondrial energy metabolism: *hsp70*, *ddit4*, *vtn*, *evpl*, and *edar*. (**C**) Core transcriptional regulatory factors: *nr4a3*, *nr4a2*, and *ebf1*. (**D**) Oxidative stress and intestinal mucosal barrier homeostasis: *acsl4*, *elovl2*, *enpp7*, and *gdf6a*. *, **, ***, and **** denote significant differences at *p* < 0.05, *p* < 0.01, *p* < 0.001, and *p* < 0.0001, respectively.

To validate the transcriptomic findings, the expression of 17 selected genes involved in inflammation, lipid metabolism, transcriptional regulation, and oxidative stress/barrier function was examined by qRT-PCR (Figure 7).

For inflammation-related genes (Figure 7A), *cxcl8* and *cxcl14* were significantly downregulated in the DNJ10 group, while *nfkb1* was significantly reduced in the DNJ5 group. No significant changes were observed for *p2rx7* or *fos*.

Regarding lipid metabolism and mitochondrial function (Figure 7B), *ddit4* and *edar* were significantly elevated in all three DNJ-treated groups, whereas *hsp70* was significantly decreased only in the DNJ15 group. *vtn* and *evpl* exhibited no significant differences among groups.

Among transcriptional regulators (Figure 7C), *ebf1* was significantly upregulated in the DNJ5 and DNJ10 groups, while *nr4a3* and *nr4a2* showed no significant changes across all treatments.

For oxidative stress and barrier homeostasis-related genes (Figure 7D), *elovl2* and *enpp7* were significantly elevated in the DNJ5 and DNJ10 groups, whereas *acsl4* and *gdf6a* showed no significant differences among groups.

## 4. Discussion

### 4.1. DNJ Dose-Dependently Remodels the Lipid Metabolic Network

High-lipid diets are widely used in intensive aquaculture to promote growth, yet long-term feeding readily induces excessive lipid accumulation, oxidative stress, and reduced disease resistance in fish [26]. 1-Deoxynojirimycin (DNJ), a characteristic bioactive alkaloid derived from mulberry leaves, has been shown to target glucose and lipid metabolic pathways and restore lipid homeostasis [12,27]. In the present study, by integrating serum biochemistry, intestinal qRT-PCR, intestinal transcriptomics, untargeted metabolomics, and gut microbiota profiling, we systematically demonstrated a dose-dependent lipid-regulatory effect of DNJ, with DNJ10 identified as the optimal dosage for mediating intestinal lipid remodeling.

Serum biochemical analysis showed that TG was significantly reduced only in the DNJ10 group, while TC were significantly decreased in both the DNJ10 and DNJ15 groups. These results are consistent with previous findings that DNJ promotes lipolysis and inhibits lipid synthesis. In the low- and medium-dose groups (DNJ5 and DNJ10), *elovl2* expression was significantly elevated. ELOVL2 is a long-chain fatty acid elongase that specifically catalyzes the carbon-chain elongation of C20/C22 polyunsaturated fatty acids (PUFAs), mediating the synthesis of functional long-chain PUFAs such as DHA and DPA, thereby optimizing the overall lipid composition [28,29]. The pro-lipotoxic gene *acsl4* exhibited a consistent downward trend across all treatment groups, which would help reduce the accumulation of deleterious lipid intermediates. ACSL4 is the rate-limiting enzyme that catalyzes arachidonic acid activation; its high expression supplies abundant substrates for lipid peroxidation, triggering lipotoxic injury and metabolic inflammation. Conversely, ACSL4 inhibition significantly reduces the accumulation of pro-oxidative PUFAs [30]. These molecular changes are consistent with findings from in vitro hepatocyte models of DNJ intervention against lipid deposition [14].

Untargeted metabolomic analysis further confirmed that the magnitude of lipid metabolic pathway perturbation peaked in the DNJ10 group, with arachidonic acid metabolism identified as the core regulatory target. Arachidonic acid is a prototypical pro-inflammatory lipid precursor; upon release, it is metabolized through three oxidative pathways—COX, LOX, and CYP450—to generate chemotactic mediators such as prostaglandins and leukotrienes, which induce the transcription of pro-inflammatory genes including *cxcl8* and *cxcl14* [31]. The simultaneous upregulation of *elovl2* and downregulation of *acsl4* by DNJ reduces the intracellular pool of activatable arachidonic acid substrates, thereby blocking the arachidonic acid oxidative cascade at its source and ultimately decreasing the production of pro-inflammatory lipid mediators and the lipid peroxidation product MDA. This achieves a dual regulatory effect of lipid lowering and suppression of metabolic inflammation.

Lipid homeostasis in the intestinal mucosa is not regulated autonomously by enterocytes alone, and this also applies to the liver, which was not sampled in the present study. The gut microbiota, as a core mediator of the gut–liver axis, can modulate intestinal and hepatic lipid metabolic and inflammatory signaling through the secretion of diverse lipid and amino acid metabolites. It should be emphasized that all transcriptomic and qRT-PCR measurements in this study were performed on intestinal tissue; the hepatic responses discussed below are therefore inferred through the gut–liver axis and the systemic biochemical read-outs, and not from direct hepatic sequencing. A key differential taxon enriched in the DNJ10 group was Ruminococcaceae. This functional bacterial family, belonging to the phylum Firmicutes, secretes extracellular lipases that promote dietary lipid breakdown and ferments to produce large amounts of short-chain fatty acids (SCFAs) such as butyrate [32]. In previous studies, SCFAs entering the liver via the portal vein have been reported to upregulate *pgc-1α* expression, expression, activate the mitochondrial fatty acid β-oxidation pathway, and simultaneously block NF-κB signaling, thereby suppressing the transcription of pro-inflammatory chemokines such as *cxcl8* and *cxcl14* [33,34].

### 4.2. DNJ Attenuates Metabolic Inflammation via Layer-Specific Pathways to Achieve Immune Homeostasis

Metabolic inflammation is driven collectively by lipid accumulation, oxidative stress, and excessive activation of the NF-κB pathway [35]. DNJ can regulate inflammatory signaling in a layer-specific manner, achieving a balanced mode of “suppressing excessive inflammation while preserving innate immunity,” with distinct anti-inflammatory pathways activated at different DNJ concentrations.

#### 4.2.1. Low-Dose DNJ: Blocking the Upstream NF-κB Inflammatory Switch

NF-κB1 is a core transcription factor mediating metabolic inflammation; its pathway activation induces the release of numerous pro-inflammatory chemokines and cytokines, including *cxcl8* and *il-6* [36]. In the present study, *nfkb1* expression was significantly downregulated in the DNJ5 group, suggesting that low-dose DNJ directly suppresses NF-κB signaling at the transcriptional level, blocking the initiation of the inflammatory cascade from upstream. Correspondingly, serum MDA content was significantly reduced in the DNJ5 group, indicating alleviation of lipid peroxidation damage and a decrease in systemic oxidative stress. This pattern is consistent with previous reports on the anti-inflammatory effects of DNJ in poultry liver [16]. Meanwhile, complement C3 and immunoglobulin IgM showed no significant changes in this group, indicating that low-dose DNJ only alleviates basal lipid-induced oxidative inflammation without activating complement-mediated innate immune pathways [22].

#### 4.2.2. Medium-Dose DNJ: Targeting Downstream Chemokines and Balancing Innate Immunity

CXCL14 mediates immune cell infiltration and dendritic cell maturation, upregulating cell surface MHC-I molecules to regulate immune cell migration and mobilization [37]. CXCL8 is a classic neutrophil chemokine; the two act synergistically to induce low-grade chronic tissue inflammation [38]. Our results showed that *cxcl8* and *cxcl14* expression was significantly downregulated in the DNJ10 group, while serum complement C3 content was markedly elevated. This suggests that DNJ10 establishes a layered immune regulatory pattern: on one hand, it suppresses neutrophil chemotaxis and reduces local inflammatory infiltration; on the other hand, it enhances complement-mediated innate immune defense [39]. This differential regulatory pattern is closely related to arachidonic acid metabolic remodeling: downstream lipid mediators of arachidonic acid can activate the NF-κB pathway, thereby promoting chemokine transcription; DNJ10 may indirectly suppress intestinal *cxcl8* and *cxcl14* expression by modulating lipid metabolic signaling, while systemic complement C3 was elevated [31]. Regarding oxidative stress indicators, the lipid peroxidation marker MDA was significantly reduced in the DNJ5 and DNJ10 groups, while T-AOC and SOD showed only slight increases without statistical significance. qRT-PCR analysis revealed that *ddit4* and *edar* were significantly upregulated in all DNJ-treated groups. DDIT4 is a key cellular stress regulator that has been reported to negatively feedback to inhibit the mTOR pathway, alleviating abnormal cell proliferation and oxidative damage [40]. EDAR is a damage-response-related gene involved in maintaining cellular homeostasis; the two act synergistically to exert auxiliary anti-inflammatory effects.

Heat shock proteins (HSPs) repair misfolded proteins, prevent the accumulation of immature polypeptides under stress conditions, alleviate proteotoxic damage, and maintain normal cellular physiological functions [41]. The endoplasmic reticulum stress marker gene *hsp70* was significantly downregulated only in the DNJ15 group, suggesting that low- and medium-dose DNJ (DNJ5 and DNJ10) induce physiological adaptive ER stress that facilitates lipid metabolic reprogramming without obvious cellular damage. After long-term high-concentration DNJ intervention, the metabolic compensatory mechanisms become more complete, ER stress levels decline, and the corresponding anti-inflammatory protective effects are attenuated.

#### 4.2.3. High-Dose DNJ: Diminished Anti-Inflammatory Efficacy

Compared with the multi-layered synergistic anti-inflammatory regulatory network of DNJ5 and DNJ10, none of the inflammation-related genes showed significant differential expression in the DNJ15 group. Combined with the gut microbiota results, high-dose DNJ induced a shift in the intestinal community toward bile-metabolizing spirochetes, with loss of SCFA-producing beneficial bacteria, thereby abolishing the microbiota-mediated anti-inflammatory pathway of the gut–liver axis. Collectively, the immune balance regulatory capacity of DNJ15 is significantly weaker than that of DNJ10, a feature fully consistent with the overall pattern of multi-omics molecular perturbation intensity gradually declining with increasing DNJ dose.

### 4.3. Gut Microbiota Remodeling Mediates the Interplay Between Lipid Metabolism and Host Inflammation

The abundance and community composition of the gut microbiota in aquatic animals are core factors regulating host growth, metabolism, nutrient absorption, and immune defense [42]. Fish gut microbiota structure is influenced by multiple factors, including host genetics, developmental stage, feeding patterns, dietary composition, and rearing environment [43]. Gradient DNJ supplementation significantly remodeled the intestinal microecological structure of sturgeon, with each dosage group enriching characteristic functional bacteria that mediate distinct lipid metabolic and inflammatory regulatory pathways, constructing a complete gut microbiota–lipid metabolism–inflammatory response regulatory axis in the host [32,34].The signature taxon of the DNJ5 group, *Cetobacterium_A*, is a classic core beneficial commensal in aquaculture that ferments to produce short-chain fatty acids and branched-chain amino acids. This bacterium can synthesize argininosuccinate to activate the enterhepatic urea cycle, enhancing amino acid transport and catabolism, with the metabolome concurrently enriching ABC transporter and amino acid metabolism pathways. On one hand, it suppresses NF-κB pathway activation locally in the intestine, reducing basal inflammation levels; on the other hand, it relies on its characteristic metabolite acetate to activate the parasympathetic nervous pathway, promoting insulin secretion, steadily regulating blood glucose, and alleviating ammonia-induced oxidative damage, achieving a mild improvement in metabolic homeostasis [44,45]. Meanwhile, the overall gut microbial diversity was greatly enhanced in the DNJ5 group, further reducing the risk of glucose and lipid metabolic disorders.

The DNJ10 group specifically enriched Ruminococcaceae and *Paludicola* from the phylum Firmicutes. Butyrate and lipolytic bioactive substances produced by Firmicutes metabolism enter the host via the enterohepatic circulation and act on the intestinal mucosa, where they upregulate the functional fatty acid synthesis gene *elovl2*, while simultaneously blocking NF-κB signaling to downregulate pro-inflammatory chemokines such as *cxcl8*/*cxcl14*. This represents a core microbial mediator with dual protective effects of lipid lowering and inflammation suppression [33].

The signature differential bacterium of the DNJ15 group was *Brevinema andersonii* from the phylum Spirochaetota. This strain only participates in intestinal bile acid cycling and conversion and cannot synthesize anti-inflammatory and pro-lipolytic metabolites such as butyrate, acetate, or argininosuccinate, making it difficult to regulate lipid accumulation and chronic inflammation. This explains, from the perspective of intestinal microecology, the intrinsic mechanism by which the comprehensive regulatory effects of high-dose DNJ are significantly weaker than those of medium-dose DNJ10 [46].

Similar dose-dependent, microbiota-mediated regulatory phenomena have also been reported in other fish species, although the relative contribution of each gut–liver axis route differs among species. In spotted sea bass (*Lateolabrax maculatus*), dietary mulberry leaf extract likewise improved antioxidant capacity, immune status, and intestinal health [22,39]; in largemouth bass (*Micropterus salmoides*), microbiota-mediated bile acid/FXR signaling was identified as a key route by which dietary additives regulate lipid metabolism [20,23]. In the present study, however, the response of Amur sturgeon was dominated by SCFA-producing Firmicutes and the downstream suppression of NF-κB-mediated chemokines rather than by bile acid receptor signaling. This contrast suggests that fish species with distinct gut architecture and microbial ecology may rely on different gut–liver communication routes, rather than sharing a single conserved regulatory mechanism.

### 4.4. Dose–Response Window of DNJ: Trade-Offs Among Growth, Metabolism, and Immunity

Growth performance, organ indices, serum biochemistry, and multi-omics data collectively demonstrate that DNJ exhibits a typical dose–response window effect: low-dose provides mild health maintenance, medium-dose achieves optimal comprehensive regulation, and high-dose causes metabolic compensation that encroaches upon growth and nutrition. The quadratic polynomial regression model further quantified the optimal supplementation range.

#### 4.4.1. Qualitative Gradient Phenotypic Patterns

DNJ5 (low dose): The mild metabolic improvement without growth suppression is mechanistically attributable to the enrichment of *Cetobacterium_A* and the marked restoration of microbial diversity, which activated basal anti-inflammatory and amino acid metabolic routes without strongly perturbing systemic lipid turnover.

DNJ10 (medium dose): The synchronous decrease in GLU, TC, TG, and MDA was mechanistically linked to Ruminococcaceae-derived butyrate, which suppressed NF-κB-mediated chemokine transcription; the moderate increases in HSI and VSI reflected reversible physiological compensation associated with lipid remodeling and complement synthesis rather than pathological damage, as ALP remained normal and no growth metric differed significantly from the control.

DNJ15 (high dose): The diminished regulatory efficacy arose from the loss of SCFA-producing taxa and the dominance of the bile-metabolizing *Brevinema*, which abolished the microbiota-mediated anti-inflammatory route of the gut–liver axis; consequently, substantial energy was diverted to glucose–lipid homeostasis compensation through bile acid pathways, reducing nutrient deposition efficiency.

#### 4.4.2. Quadratic Regression Quantification of the Optimal Dose

Quadratic polynomial regression models are widely used in aquatic nutrition to quantify the optimal supplementation range of functional additives, and joint fitting across multiple physiological indicators can precisely identify the optimal dosage window [47,48]. In the present study, this approach was applied to four physiological indicators—GLU, TG, MDA, and HSI—to determine the optimal dietary DNJ inclusion level for Amur sturgeon.

Direct dietary DNJ supplementation data in aquatic animals are currently scarce. To our knowledge, the only published study using purified DNJ as a feed additive in an aquatic animal is in Chinese giant salamander (*Andrias davidianus*), where 40 mg/kg DNJ for 90 d significantly improved growth performance, liver antioxidant capacity, and immune function [18,49]. In juvenile tilapia, DNJ from *Bacillus subtilis* improved antioxidant and antibacterial activities at the cellular level (5 mg/L), but no dietary inclusion level was reported [50]. Given the absence of systematic dose–response data for DNJ in fish, three graded levels (DNJ5, DNJ10, and DNJ15) were designed to cover a low-to-high range and to establish a dose-dependent relationship in *A. schrenckii*.

Quadratic polynomial regression fitting of the four indicators yielded the following results: the theoretical minimum for TG corresponded to a supplementation concentration of 8.48 mg/kg, the theoretical minimum for GLU to 7.89 mg/kg, the theoretical minimum for MDA to 8.06 mg/kg, and the theoretical peak for HSI to 8.16 mg/kg. The optimal concentrations for all four indicators were highly convergent, all falling within the DNJ10 supplementation range. The mathematical fitting results mutually corroborate the multi-omics and physiological-biochemical phenotypes: at the medium concentration, lipolysis and anti-inflammatory pathways were synchronously activated, and glucose–lipid disorders and oxidative damage were minimized. The liver exhibited reversible physiological compensation due to lipid metabolic remodeling and enhanced complement synthesis. Beyond the optimal dosage window, the organism-initiated bile acid compensatory pathways, with large amounts of energy expended to maintain metabolic homeostasis, hindering growth and nutrient deposition.

Consistent with this interpretation, independent support for the optimal-dose window comes from the non-monotonic metabolomic response: the number of differential metabolites peaked at DNJ10 (995 metabolites), compared with 329 in DNJ5 and 377 in DNJ15. This pattern is consistent with the transcriptomic data (176 DEGs at FDR < 0.05 in DNJ10 vs. 76 in DNJ5 and 62 in DNJ15) and with the physiological read-outs (lowest GLU, TG, and MDA; highest HSI). If the optimal dose were simply the lowest dose, metabolomic perturbation should decrease monotonically with increasing DNJ; instead, it peaks at the medium dose, exactly where the quadratic regression of GLU, TG, MDA, and HSI converges (7.89–8.48 mg/kg). This convergence across metabolomic, transcriptomic, and physiological layers reinforces the interpretation that DNJ10 lies within the optimal dosage window.

This low-dose-promotion, high-dose-inhibition dose–response pattern is characteristic of the hormetic effects typical of plant-derived bioactive substances [51].

## 5. Limitations

Several limitations of this study should be acknowledged. First, liver tissue was not sampled; the hepatic arm of the gut–liver axis was inferred from intestinal transcriptomics and systemic biochemical read-outs rather than directly validated. Second, key microbial metabolites such as SCFAs and bile acids were not quantified. Finally, the observation period was limited to 8 weeks, and only one developmental stage was examined.

## 6. Conclusions

In summary, our multi-omics integrative analysis demonstrates that DNJ exerts a dose-dependent regulatory pattern on intestinal lipid metabolism and metabolic inflammation in sturgeon: low-to-medium doses are effective, whereas the effects diminish or reverse at the high dose, as reflected in the weakened anti-inflammatory response and impaired growth of the DNJ15 group. Among the doses tested, the medium dose (DNJ10) achieved the best balance between metabolic benefits and growth performance. Because quadratic regression of the key indicators converged on an estimated optimum of 8.0–8.5 mg/kg, DNJ10 should be regarded as the experimental dose closest to—rather than identical with—this regression-derived optimum. The gut microbiota–liver axis serves as a key mediator of these effects. From the perspective of the aquaculture industry, these results indicate that supplementing high-lipid feeds with approximately 8.0–8.5 mg/kg DNJ (practically approximated by the DNJ10 level) is a feasible nutritional strategy to mitigate lipid metabolic disorders and metabolic inflammation in intensive sturgeon farming, with direct implications for feed formulation, fish health management, and the sustainability of caviar-oriented production.

## Data Availability

Dataset available on request from the authors.

## Supplementary Materials

The following supporting information can be downloaded at: https://www.mdpi.com/article/10.3390/biology15201809/s1. Table S1: Formulation of experimental diets (%). Table S2: The complete calculation formula. Table S3: Primer sequences for real-time PCR. Table S4: Effects of DNJ on the growth performance and morphometric parameters in *Acipenser schrenckii*.

## Abbreviations

The following abbreviations are used in this manuscript:

DNJ	1-deoxynojirimycin
TC	Total cholesterol
TG	Triglyceride
GLU	Glucose
T-AOC	Total antioxidant capacity
TP	Total protein
SOD	Superoxide dismutase
MDA	Malondialdehyde
C3	Complement 3
C4	Complement 4
IgM	Immunoglobulin M
ALP	Alkaline phosphatase
*cxcl8*	C-X-C motif chemokine ligand 8
*cxcl**14*	C-X-C motif chemokine ligand 14
*edar*	Ectodysplasin A receptor
*evpl*	envoplakin
*ebf1*	early B-cell factor 1
*elovl2*	ELOVL fatty acid elongase 2
*enpp7*	Ectonucleotide pyrophosphatase/phosphodiesterase 7
*gdf6*	Growth differentiation factor 6
*hsp70*	Heat shock protein 70
*nr4a2*	Nuclear receptor subfamily 4 group A member 2
*nr4a**3*	Nuclear receptor subfamily 4 group A member 3
*p2rx7*	Purinergic receptor P2X 7
*pufas*	Polyunsaturated fatty acids
*scfas*	Short-chain fatty acids
*ddit4*	DNA damage inducible transcript 4
*vtn*	Vitronectin
*gdf6a*	Growth differentiation factor 6a
