# Supplementary material for: Multi-Omics Reveals Dose-Dependent Modulation of Intestinal Lipid Metabolism and Metabolic Inflammation by 1-Deoxynojirimycin in Amur Sturgeon (Acipenser schrenckii)
Source: Biology (Basel). 2026 Oct 10;15(20):1809. doi: 10.3390/biology15201809 (PMC13701734; doi:10.3390/biology15201809)
Supplement: Supplementary file 1 [file biology-15-01809-s001.zip › biology-4590542-supplementary.pdf]

Supplementary Files

Table S1. Formulation of experimental diets (%).

Ingredients	CK	DNJ5	DNJ10	DNJ15
Fish meal[1]	55.00	55.00	55.00	55.00
Wheat middling	18.00	18.00	18.00	18.00
Wheat gluten meal	19.00	19.00	19.00	19.00
Fish oil	3.50	3.50	3.50	3.50
Soybean oil	3.00	3.00	3.00	3.00
L-Lysine	0.05	0.05	0.05	0.05
Yeast extract	1.00	1.00	1.00	1.00
DL-Met	0.00	0.00	0.00	0.00
$Ca(H_2PO_4)_2$	0.05	0.05	0.05	0.05
Thr[2]	0.00	0.00	0.00	0.00
Mineral premix	0.20	0.20	0.20	0.20
Vitamin premix	0.20	0.20	0.20	0.20
Total	100	100	100	100
DNJ (mg/kg)	0	5	10	15

Table S2. The complete calculation formula.

Items	Calculation formula
WGR(%)	100×(Wt -Wo) /Wo
SGR(%/day)	100×[(ln(Wt)-ln(Wo)] /days of feed test
FCR	feed intake/(Wt -Wo + dead experimental fish weight)
FR(%)	100×total dry matter intake of feed/[(Wt+Wo)/2]/days of feed test
SR (%)	100×terminal quantity/original quantity
CF (g/cm^3)	100×(body weight) /[(body length)^3]
HSI (%)	100×(hepatic weight) / (body weight)

VSI (%)	100×(viscus weight) / (body weight)

Wt is the terminal body weight, Wo is the original body weight, the same below.

Table S3. Primer sequences for real-time PCR.

Gene	Sequence 5′-3′	Target size (bp)	Tm (°C)
βactin	F: CGGTTTCGCTGGAGATGATG<br>R: TCAGTGAGCAGGACGGGGTG	135	60
cxcl8	F: CATCCCCAGAGGATCACACT<br>R: CCAGGGTGCACGAAGATTCA	90	58-59
cxcl14	F: GACGTGCAGAAGCTGGAGAT<br>R: AGTACTCCTGGCCCTTGAAG	102	58-59
nfκb1	F: CAGGGGGAGAGGAGATGTAT<br>R: AGAGTGGGCCATTCTCATCT	92	57-58
p2rx7	F: GTGCGTGTTCAGAGGAACAC<br>R: TGGTGCTACTGTGGAAGGAC	86	57-58
fos	F: GCTCCCAGTTCTCAGATCCA<br>R: CAGAGCTGGTGCAAAAGCTG	99	58-59
hsp70	F: GATATGAAGCACTGGCCGT<br>R: GACACTTTCTGGCCCAGGT	164	58-59
ddit4	F: GCTCTGAAGTGGTGGACCT<br>R: GCATCTGACTGCTCAACACA	190	58-59
vtn	F: GTTGCTGCGTGGACTATGAC<br>R: TCTGGGTCTGACAGCGTTG	179	57-59
edar	F: AGGCTATGACTGCATGCCT<br>R: TGGCACACCATCCCATAGATG	207	58-59
nr4a3	F: ACGCAGGGGACTATAGCTC<br>R: CGGAGGACTGCATCTGGTAC	183	58-60
nr4a2	F: CTGAGATCACAGCCACCAC<br>R: GCAGGTGGCTTTGTTGGTG	174	58-60
ebf1	F: AGCGAGGGATGGACTACAG<br>R: CTGCTTGGACTTGTAGGACAG	192	57-58
acsl4	F: TAAGTGTGTGCTGTCGGAGC<br>R: GATCCGTGGGGACAGGTC	179	58-59
elovl2	F: GAAGCGGACACAAGGGTTG<br>R: CTTTGTCCACAGGGTATCCAG	188	57-58
enpp7	F: CGAATCTGGATGCCATGGC<br>R: TGACAAACTGTGTGCGGTAG	192	58
gdf6a	F: GATCATAGCGCCACTGGACT	90	58-59

R: AGCGTGGTTTGTTGGCTCTA

Tm: melting temperature; F: forward primer; R: reverse primer.

Table S4. Effects of DNJ on the growth performance and morphometric parameters in *Acipenser schrenckii*.

	CK	DNJ5	DNJ10	DNJ15
Growth performance				
IBW(g)	162.91±0.67	163.47±0.51	163.79±0.96	164.16±0.41
FBW(g)	362.92±3.24	365.43±4.30	335.78±2.28	334.86±9.75
WGR(%)	122.81±2.89	123.57±3.17	105.01±0.99	103.96±5.63
SR(%)	95.56±1.81	95.56±1.81	95.56±3.63	95.56±3.63
SGR(%/d)	$1.14±0.02^{ab}$	$1.17±0.03^{a}$	$1.03±0.01^{ab}$	$1.02±0.04^{b}$
FCR	$0.84±0.02^{b}$	$0.84±0.02^{b}$	$0.95±0.03^{ab}$	$0.97±0.02^{a}$
FR(%)	0.76±0.02	0.75±0.02	0.78±0.05	0.88±0.08
Morphometric parameters				
CF(g/cm^3)	0.71±0.02	0.75±0.02	0.78±0.01	0.71±0.02
HSI(%)	$2.22±0.04^{b}$	$2.26±0.05^{ab}$	$2.49±0.08^{a}$	$2.21±0.07^{b}$
VSI(%)	$7.13±0.10^{b}$	$7.27±0.11^{ab}$	$7.83±0.22^{a}$	$7.29±0.15^{ab}$

$^{a-b}$ Within a row, means without a common superscript letter differ at $p < 0.05$, $n = 3$.
